# Supplementary material for: Effectiveness and costs associated with a lay counselor–delivered, brief problem-solving mental health intervention for adolescents in urban, low-income schools in India: 12-month outcomes of a randomized controlled trial
Source: PLoS Med. 2021 Sep 28;18(9):e1003778. doi: 10.1371/journal.pmed.1003778 (PMC8478208; doi:10.1371/journal.pmed.1003778)
Supplement: S1 Protocol — (PDF) [file pmed.1003778.s001.pdf]

The effectiveness of a low-intensity, lay counsellor-delivered, problem-solving intervention for common mental health problems in school-based adolescents in New Delhi, India: PRIDE randomized controlled trial – Statistical Analysis Plan for 12-month follow-up

Version 1

28/01/20

Investigators:
Vikram Patel, Daniel Michelson, Kanika Malik, Rooplata Sahu, Aoife Doyle, Paulomi Sudhir, Michael King, Pim Cuijpers, Helen A. Weiss, Giulia Greco, Bruce Chorpita and Chris Fairburn

Study Coordinator
Kanika Malik

Data manager
James E.J

Study statisticians
Aoife Doyle
Helen Weiss

Original Trial registration numbers
NCT03630471

**Role:**	**Trial statistician**
**Name:**	**Helen Weiss**
**Signature:**	[illegible]
**Date:**	**Feb 2nd 2020**

CONTENTS

1. Purpose and scope ... 3
2. Description of the main study & additional study ... 3
2.1 Principal research objectives ... 4
2.2 Study design (12 month) ... 6
2.3 Time of outcome assessment ... 8
2.4 Serious Adverse Events (SAEs) ... 8
2.5 Process evaluation ... 9
2.6 Data management ... 9
3. Variables ... 10
3.1 Baseline variables ... 10
3.2 6- and 12-week variables ... 10
3.3 Outcome variables ... 10
3.4 Process variables (in both arms) ... 11
3.5 Serious Adverse Events (SAEs) ... 11
3.6 Potential effect moderators ... 11
3.7 Potential effect-mediators ... 11
4. Data analysis plan ... 11
4.1 Recruitment and representativeness of recruited participants and participants who completed follow up ... 11
4.2 Loss to follow-up and other missing data ... 12
4.3 Adverse event reporting ... 12
5. Outcome analysis ... 12
5.1 Main analysis of intervention differences ... 12
5.1.1 Analysis of primary outcomes at 12 months ... 12
5.1.2 Analysis of secondary outcomes over 12 months ... 13
5.2 Statistical considerations ... 13
5.3 Planned sub-group (moderator) analyses ... 13
5.4 Mediation analyses ... 13
5.5 Adherence analysis ... 14
5.6 Additional analyses ... 14
6. References ... 15
7. Appendix I: Dummy tables ... 16
Table A1 Baseline characteristics of completers of outcome evaluation and participants lost to follow-up at 12-month time point (LTFU) ... 16
Table A2. Primary outcomes according to baseline characteristics in the intervention arm 18
Table A3. Process indicators for trial participants in the PRIDE and EUC arms ... 23
Table A4: Intervention effect on SDQ total and YTP mean scores at 12 months ... 19
Figure 2. Mean SDQ total difficulties score over time according to arm ... 19
Figure 3. Mean YTP mean score over time according to arm ... 19
Table A5. Primary Outcome by potential effect modifiers: SDQ total difficulties scores at 12 months ... 20
Table A6. Primary Outcome by potential effect modifiers: YTP scores at 12 months21
Table A8: Mediation effect of Perceived stress measured at 6 weeks ... 23
Table A9: Mediation effect of frequency of POD booklet use in the past 12 months24
Table A10: Mediation effect of frequency of use of techniques learnt in the past 12 months 24
Table A11. Secondary outcomes: Intervention effect over 12 months ... 25

1. Purpose and scope

The purpose of this document is to describe procedures and considerations for analysis of data from the PRIDE trial in India. This analysis plan covers the additional 12-month follow-up analysis only. The 6 and 12 weeks analyses, as outlined in the study protocol, is covered by a separate analysis plan https://clinicaltrials.gov/ProvidedDocs/71/NCT03630471/SAP_000.pdf. The initial analyses have been completed and presented during the TSC/DSMB meeting on May 20, 2019.

The findings from 6 and 12 weeks analyses indicated that participants both from the Intervention & Enhanced Usual Care (EUC) control condition continued to improve on mental health symptoms and idiographic problems from primary (6 weeks) to secondary assessment (12 weeks) and there was widening of gap in mean SDQ scores & YTP scores between two arms at 12 weeks. Based on the recommendations of the TSC/DSMB, it was decided to conduct an additional follow-up to examine the longer-term effect of the intervention on symptoms and problems.

2. Description of the main study & additional study

Main study:

The goal of the trial was to evaluate the effectiveness of a low-intensity, lay counsellor-delivered, problem-solving intervention for adolescents with common mental health problems attending Government-run secondary schools in New Delhi, India.

The two-arm, parallel-design, individually randomised controlled trial was conducted in six Government-run secondary schools from the National Capital Territory of Delhi, India. The schools were purposively selected in consultation with the Department of Education, Government of New Delhi, India. This includes five same-sex schools (three boys' schools and two girls' schools) and one mixed school. The eligibility criteria for the main study are given in Box 1.

Box 1 Trial eligibility criteria

Eligible adolescent participants will be:

i) enrolled as a student in Grades 9-12;
ii) aged 13-20 years;
iii) experiencing elevated mental health symptoms, based on response in the borderline or abnormal range of the self-report SDQ Total Difficulties Score $\geq$19 for boys and $\geq$20 for girls (derived from a normative reference sample of 1087 students (M age=16.4 years) from urban India)
iv) experiencing significant distress and/or functional impairment, based on response in the abnormal range (>=2) on the self-reported Impact Supplement of the SDQ;
v) experiencing difficulties for >1 month, based on response to the self-reported chronicity item of the Impact supplement of the SDQ.
vi) able to provide informed consent (or assent if under 18 years, supported by parental consent) to participate.

Eligible caregiver participants will be:

i) a primary parental caregiver or guardian for the index adolescent; and
ii) able to provide informed consent for their and index adolescent's participation (if under 18 years);
iii) if adolescent age 18 or more years, caregiver involvement is in turn subject to the index adolescent's preference.

A total of 251 participants were recruited in the main study. The key trial design details including randomisation, sample size estimation, duration of intervention period, enhanced usual care, window of follow-ups, and data management, are described in the protocol publication.[1]

Additional study:

The goal of the additional study is to evaluate the longer-term impact of a low-intensity, problem-solving intervention in reducing adolescent-reported mental health symptoms and idiographic problems for adolescents with common mental health problems who participated in the PRIDE randomized control trial.

2.1 Principal research objectives

The primary objective of the additional study is to evaluate the impact of a low-intensity, problem-solving intervention (intervention arm) in reducing adolescent-reported mental health symptoms and idiographic problems at 12 months.

Secondary objectives are:

- To evaluate the impact of the intervention on adolescent-reported distress/functional impairment, perceived stress, and mental wellbeing over 12 months.
- To explore whether a theoretically-informed a priori factor (perceived stress at 6 weeks) mediates the effects of the intervention on symptoms of mental health difficulties and idiographic problems at 12 months

The primary hypothesis is that the intervention will be superior to an Enhanced Usual Care (EUC) control condition in reducing the severity of adolescent-reported mental health symptoms and idiographic problems at 12 months post-randomisation.

The secondary hypotheses are that the intervention will be superior to the control condition with respect to the following outcomes, over a 12-month period post-randomisation

1. Reducing self-reported adolescent mental health symptoms and idiographic problems;
2. Reducing self-reported distress/functional impairment;
3. Reducing self-reported perceived stress;
4. Improving self-reported adolescent wellbeing
5. Improving remission, derived from the ‘crossing clinical threshold’ method applied to self-reported adolescent mental health symptoms and associated distress/functional impairment

Tables 1-2 provides a summary of primary and secondary outcomes.

Table 1 Primary outcomes

Measures	Description	Primary outcomes at 12 months post-randomisation
Strengths and Difficulties Questionnaire (SDQ) Total difficulties score	25-item self-report measure of youth mental health difficulties (Goodman et al., 2000). A Total Difficulties scale score is derived by summing items from four problem subscales (Emotional, Conduct, Hyperactivity/inattention, and Peer relationship), while a fifth subscale measures prosocial functioning and does not contribute to the overall severity score. Individual problem scale items are scored from 0-2 (with higher scores indicating greater problem severity), giving a range of 0-40 for Total Difficulties.	Self-reported total difficulties score
Youth Top Problems (YTP)	The Youth Top Problems (YTP) is a brief, idiographic measure which identifies, prioritizes and scores respondents' three main problems (Weisz et al., 2011). Each nominated problem is scored from 0 ('not a problem') to 10 ('huge problem'). A mean severity score is calculated by summing individual problem scores and then dividing by the number of nominated problems.	YTP severity score

Table 2 Secondary outcomes

Measures	Description	Secondary outcomes over 12 month period post-randomisation[a]
Strengths and Difficulties Questionnaire (SDQ) Total difficulties score	(see Table 1)	Self-reported total difficulties score
Youth Top Problems (YTP)	(see Table 1)	YTP severity score
SDQ Impact Supplement	The SDQ Impact Supplement measures distress and functional impairment associated with index mental health difficulties identified on the main SDQ scale (Goodman et al., 2000). One item on overall distress and four items on domain-specific functional impairment (home life, friendships, classroom learning, leisure activities) are individually scored from 0-2 (with higher scores indicating greater impact), generating a total impact score from 0 to 10.	Self-reported total impact score
SDQ internalising subscale	Peer and emotional sub-scales	Self-reported score
SDQ externalising subscale	Conduct and hyperactivity sub-scales	Self-reported score
Perceived Stress Scale-4-item version (PSS-4)	The PSS-4 will be used to measure the perception of stress, reflecting the degree to which situations are appraised as stressful during the preceding month (Cohen et al., 1983). This brief instrument uses a five-point scale (0=never, 1=almost never, 2 sometimes, 3=fairly often, 4=very often) to assess how often the respondent has experienced primary appraisals of events as stressful. The total score ranges between 0 and 16, with higher scores indicating a stronger tendency towards stressful appraisals.	Self-reported total score

Short Warwick-Edinburgh Mental Wellbeing Scale (SWEMWBS)	The SWEMWBS will be used to measure mental wellbeing (Stewart-Brown et al., 2009). The SWEMWBS is a unidimensional scale that comprises 7 items scored on a five-point scale (1=None of the time, 2=Rarely, 3=Some of the time, 4=Often, and 5=All of the time), with a total range from 7-35 and where higher scores indicate more positive mental wellbeing.	Self-reported total score
Remission	Remission is defined as falling below baseline eligibility cut-offs on both reported SDQ Total Difficulties score (i.e. < 19 for boys & < 20 for girls) and SDQ Impact score (< 2) at the 12 month time point.	Self-reported (based on SDQ)
Sustained remission [a]	Sustained remission is defined as falling below baseline eligibility cut-offs on both reported SDQ Total Difficulties score (i.e. < 19 for boys & < 20 for girls) and SDQ Impact score (< 2) at all three time points: 6 weeks, 12 weeks, and 12 months	Self-reported (based on SDQ)

[a] Repeated measures analysis of 6-week, 12-week, and 12-month endpoints, adjusting for baseline values (see section 5.1.2)

Table 3 Exploratory outcomes

Measures	Description	
Academic outcome 1	Examination score (%)	Self-reported score in most recent examination (March 2019)
Academic outcome 2	Proportion who cleared annual examination (number who cleared annual examination/ number of students with examination results)	Self-reported success in most recent examination (March 2019)

2.2 Study design (12 month)

The additional follow up at 12 months will be offered only to those participants who, at the time of enrolment in the study, gave consent for contact by the research team beyond the originally stipulated final end-point at 12 weeks. Those who withdrew their participation from study at any of the previous endpoint will not be contacted for the 12 month follow up. The relevant participants will be contacted by telephone. Participants who cannot be reached after four attempts will be considered lost to follow-up. If contact is made, then the index adolescent (and a caregiver if the adolescent is aged under 18 years) will be provided with verbal information about the purpose of the longer-term follow-up. If verbal assent is forthcoming, then the 12-month outcome assessments will be scheduled at participants' homes or other convenient locations, within a maximum period of 14 calendar days from the due date. Written assent/consent will be obtained in person (supported by the aforementioned printed materials) immediately prior to completing the assessments.

The Figure 1 shows the flow of participants from eligibility to primary, secondary and 12-month endpoints of assessment

Figure 1: PRIDE trial flow chart

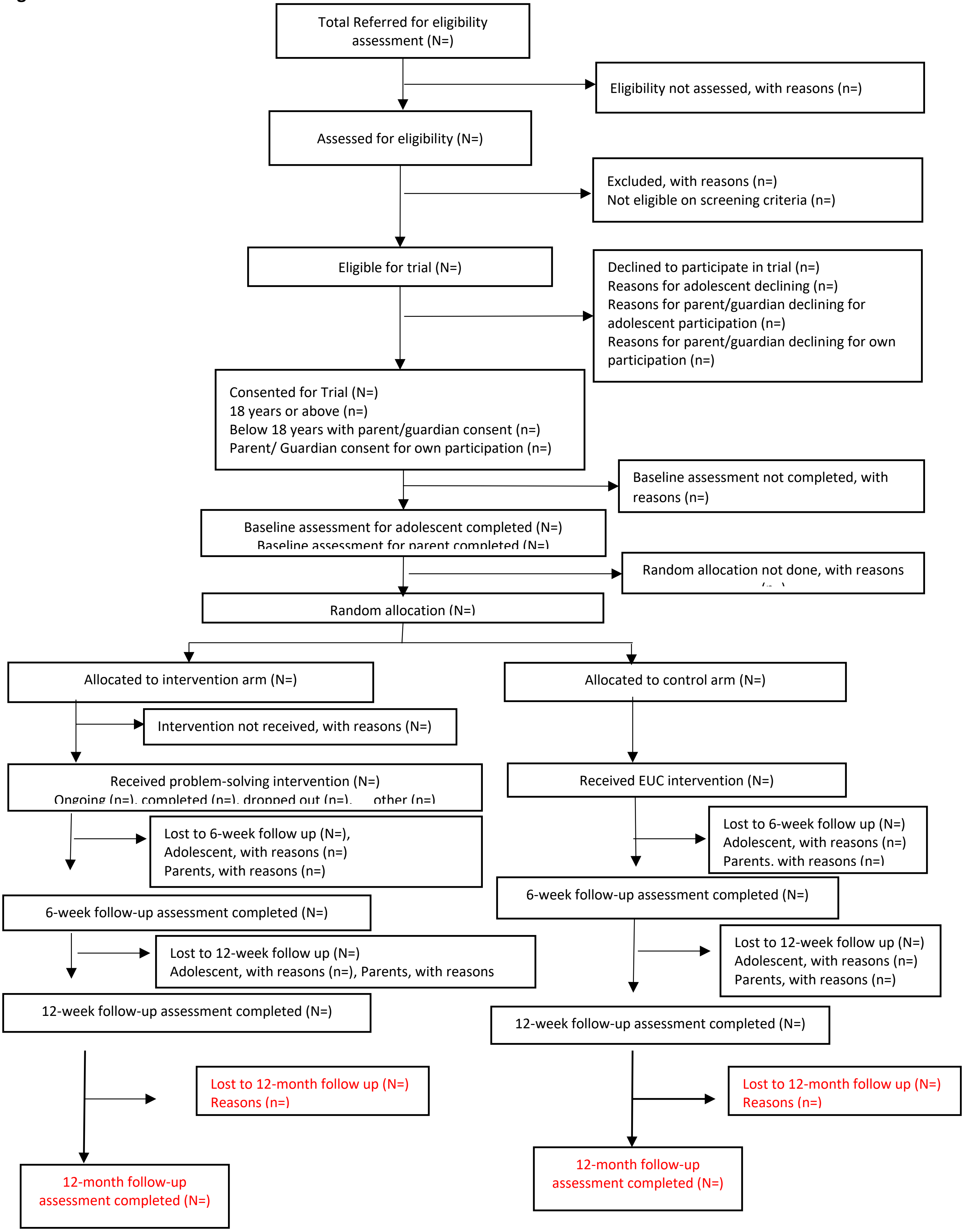

2.3 Time of outcome assessment

The primary and secondary endpoints of the main study were at 6 and 12 weeks. In this study, we will estimate the effect of the intervention at 12-month follow-up period to evaluate the longer-term effect of the intervention. A 2-week period will be allowed for the outcome assessment at 12 months (i.e. from 12 months after randomisation to 14 days after the scheduled visit date) to enable follow-up of participants some of who may be hard to reach due to long gap between the previous and current end point. For each scheduled contact, researchers will make up to four approaches, including telephonic contact and subsequent face-to-face contact at the adolescent's home to fix appointments for the assessments.

The median and interquartile range of the timing of the 12-month visits relative to the date of randomisation will be reported, along with the number and proportion of participants who were visited outside of the protocol-defined windows. We will conduct primary and sensitivity analyses as follows:

Table 4. Analysis windows to be used for the trial

Definition	12 month follow up
Primary analysis	Planned at 12 months or 365 days, [2 weeks after planned follow-up]
Sensitivity analysis	Planned at 12 months or 365 days, [4 weeks after planned follow-up]

All times relate to time after randomisation. If there was more than one visit within a window then we will refer to the visit closest to the nominal visit, with preference for earlier. For analysis, we will perform calculations in days.

For the primary analysis, the same width of window of 2 weeks as used for 6 and 12 weeks, will be applied for the 12-month visits (namely 0 weeks before and 2 weeks after). The sensitivity analysis windows will be longer (namely, 0 week before and 1 month after). These windows will cover those defined by the trial protocols.

Follow-up assessments will not take place if the trial participant is lost to follow-up or withdraws from the trial and explicitly asks not to be followed-up for outcome assessment.

2.4 Serious Adverse Events (SAEs)

SAEs include death, life-threatening event, clinical deterioration requiring hospitalization or other specialist intervention, victimization, sexual abuse, and chronic absenteeism and/or drop-out from school. Immediate safeguarding actions will prioritize the safety of participants. This may involve suicide risk assessment, informing stakeholders, facilitating intervention with specialists, and statutory reporting in line with relevant legislation, such as the Protection of Children from Sexual Offences Act 2012 and the Juvenile Justice (Care and Protection) Act 2000 (last amended in 2015).

SAEs will be reported spontaneously by adolescents/caregivers and may also be picked up by researchers or intervention providers at any contact with the participant. If a SAE is suspected, participants will be referred to a supervisor who completes a standard form. Each potential SAE will also be assessed for causality by two clinically qualified co-investigators (KM, DM) and classified as unrelated, unlikely, possible, probable or definitely related to trial participation. In the event that consensus is not reached, a third clinical psychologist (independent of the trial) will review the SAE report. Where causality is deemed to be anything other than unrelated to trial participation, the DSMC will advise on further actions such as withdrawal of individual participants, modifications to the trial protocol, continuing without modifications, or suspending/terminating the trial.

2.5 Process evaluation

We will undertake descriptive statistical analysis of quantitative process data in order to explore the differential use of intervention procedures. This will include participants' reported data on use of booklets, use of techniques learnt in program and helpfulness of techniques; and their association with the outcomes. In addition, thematic analysis will be used to code and organise qualitative written feedback on intervention satisfaction (assessed at 12-month follow-up) & qualitative interview conducted with subsample of 30 participants. The subsample will be selected purposively based on allocation status within the trial, clinical status (i.e. fully/partially/non-remitted) and school. Interviews will be transcribed for analysis.

Findings from the various data sources will be triangulated and will be used to facilitate interpretation of the main trial results. The trial statisticians may conduct further analyses to test hypotheses generated from integration of the process evaluation and trial outcome data.

2.6 Data management

Both quantitative and qualitative data will be collected. All the measures, with the exception of the YTP and referral information, will be administered via a tablet computer. Time stamps for all recruitment and outcome assessment processes will be recorded to monitor the progress of the research.

These data will be remotely uploaded as comma-separated values (CSV) files on the main data server using the customized STAR software program (OPSPL, 2013), which is compliant with Good Clinical Practice (including date and time stamps for original data entry, and an audit trail documenting any subsequent changes). The paper-based data for YTP will be entered using Epi-info database. Participant contact details and assent/consent information will be collected using paper forms and will be marked with the appropriate trial ID before being filed in separate locked cabinets. Intervention process data will be collected in paper form; these will be manually entered and stored as CSV files.

Range and consistency checks will be performed at weekly intervals separately for each data source, with all inconsistencies logged to maintain an audit trail. Identified queries will be resolved promptly by the Trial Management Committee, and the database updated accordingly. All data will be kept in separate databases and only merged into a master database after data collection had been completed and each individual database has been locked. All data will be backed-up on external hard disks on a daily basis. Access to pre-locked data will be password-protected at multiple levels and no member of the trial team apart from the data manager and independent statistician will have access to these passwords. After the dataset is locked, it will remain password-protected and trial investigators will have access to the datasets. Consent procedure, baseline and follow up assessments and intervention sessions will be audio recorded. Audio recordings will be linked with the trial ID and stored in a secure, password-protected folder. For all data, a separate file linking names and trial IDs will be kept and password-protected.

Qualitative interviews will be recorded on audio devices. Completed recordings will be uploaded onto password-protected computers and deleted from recording devices once transcribed. Efforts will be made to upload and transcribe recordings as soon as possible after interviews have been completed. In order to preserve anonymity, participant numbers will be assigned to each participant to identify the interview transcript, and pseudonyms will be used in interview transcripts where participants mention names, places or any other information that could be used to identify them.

3. Variables

3.1 Baseline variables

From refusers:

- Age
- Gender
- Class
- YTP, PSS-4, SWEMWBS
- SDQ, SDQ-impact (from eligibility screening)
- Reason for refusal

From consented participants:

- Age
- Gender
- Class
- YTP, PSS-4, SWEMWBS
- SDQ, SDQ-impact (from eligibility screening)

3.2 6- and 12-week variables

- SDQ total difficulties score
- YTP severity score
- SDQ impact
- SDQ internalising subscale
- SDQ externalising subscale
- YTP severity score
- PSS-4
- SWEMWBS
- Remission
- Intervention compliance (non-compliers 0-3 sessions; compliers 4+ sessions)
- POD booklet use in first 6 weeks of follow-up

3.3 Outcome variables

These are listed in Tables 1-3 and below:

Primary (at 12-months post randomisation; adolescent-reported)

- SDQ total difficulties score
- YTP severity score

Secondary (over 12 months of follow-up post randomisation; adolescent-reported)

- SDQ total difficulties score
- SDQ impact
- SDQ internalising subscale
- SDQ externalising subscale
- YTP severity score
- PSS-4
- SWEMWBS
- Remission
- Sustained remission

Exploratory (at 12 months of follow-up post randomisation; adolescent-reported)
- Academic outcome 1: Examination score (score in most recent examination (March 2019))
- Academic outcome 2: Proportion who cleared annual examination (number who cleared annual examination/ number of students with annual examination results (March2019))

3.4 Process variables (in both arms)
- Frequency of POD booklet use in last one year (adolescent report at 12 months)
- Frequency of use of techniques that were learnt in program in last one year (Adolescent report at 12 months)
- Helpfulness of techniques/lessons that were learnt in program in last one year (Adolescent report at 12 months)

3.5 Serious Adverse Events (SAEs)
- Death of the participant
- Life-threatening event
- Clinical deterioration requiring hospitalization or other specialist treatment
- Victimization (reported violence against the participant)
- Sexual abuse
- Chronic absenteeism and/or dropping out from school

3.6 Potential effect moderators
- Baseline chronicity of mental health difficulties (from eligibility screening; <=12 months, >12 months)
- Baseline severity of mental health difficulties (from eligibility screening; borderline or abnormal)
- YTP type (syndromal, functional, both)
- SDQ caseness profile (elevated internalising sub scale; elevated externalising subscale; elevated internalising AND externalising subscales; neither subscale elevated)

3.7 Potential effect-mediators
- Perceived stress (measured at 6 weeks)
- Frequency of POD booklet use in the past year
- Frequency of use of techniques that were learnt on the program in the last year

4. Data analysis plan

Analyses will follow CONSORT guidelines for parallel-group randomised trials.[2] Analyses for primary & secondary endpoints in the main study have been completed (and manuscripts under preparation), following recommendations from the TSC/DMSC on 20th May 2019. Analyses for this additional study will be unblinded. Analyses will be conducted in Stata version 15. Analyses will only be conducted after finalisation of the data analysis plan.

4.1 Recruitment and representativeness of recruited participants and participants who completed follow up

The trial flowchart will be expanded to include the number of students who completed the 12 month assessments and the number refusing or excluded (with reasons), actively withdrawing, and passively lost to follow-up will be shown by arm. These will be summarised by means (standard deviation), medians (interquartile range) or numbers and proportions as appropriate by key relevant subgroups (defined by gender, baseline severity of mental health difficulties, baseline chronicity of mental health

difficulties, YTP type). For continuous outcomes, histograms within each arm will be plotted to assess normality and whether transformation is required.

Initial analyses will compare baseline characteristics of participants who did and did not complete outcome assessments at 12 months, compared using Mann-Whitney tests or t-tests for continuous variables and chi2 tests for categorical variables, appropriately categorised as necessary. The variables that will be summarised are as shown in table A1 of section 8.

4.2 Loss to follow-up and other missing data

The numbers and proportions actively withdrawing from the trials and passively lost to follow-up will be reported overall and by arm for 12 months follow up. The reasons for withdrawal from the trials will be summarised.

4.3 Adverse event reporting

SAEs will be reported as the number and proportion of individuals with each type of SAE (as described above), and for any SAE, by arm. If there are a sufficient number of these, the risks and 95% CIs will be estimated and compared between intervention arms. Other (non-SAE) AEs will be reported similarly.

5. Outcome analysis

The primary analyses will be on an intention-to-treat basis at the 12-month end-point, adjusted for baseline values of the outcome measure, school (as a fixed effect in the analysis) to allow for within-school clustering, counsellor variation (as a random effect), and variables for which randomisation did not achieve reasonable balance between the arms at baseline, or those associated with missing outcome data [3]. Analyses of outcomes will be conducted using linear mixed-effects regression models for continuous outcomes with normally-distributed errors (e.g. SDQ Total Difficulties score) and generalized (logistic) mixed-effects regression models for binary outcomes (e.g. remission). Intervention effects will be presented as adjusted mean differences and effect sizes (ES), defined as standardized mean differences, with 95% confidence intervals (CIs) for continuous outcomes, and adjusted odds ratios with 95% CIs for binary outcomes.

Repeated measures analysis will be used to analyse the three follow-up time points (6 weeks, 12 weeks, 12 months). Initial models will include an interaction effect between arm and time to allow for differential effects at the two end-points. This will be retained if there is evidence of effect modification by time. No interim analyses of outcomes will be undertaken.

5.1 Main analysis of intervention differences

The outcome measures will be summarized at baseline and the 12-month follow-ups by arm, summarized by means (standard deviation), medians (interquartile range) or numbers and proportions as appropriate. For continuous outcomes, histograms within each arm will be plotted to assess how closely the scales follow a normal distribution to determine how to describe the outcomes and choice of inferential analysis method.

5.1.1 Analysis of primary outcomes at 12 months

The intervention effect on SDQ total score and YTP score will be reported as standardized mean differences (SMD; effect size), with 95% confidence intervals (CI). Linear mixed-effects regression will be used, adjusting for baseline SDQ score, school as a fixed effect, and counsellor variation as a random effect. Adjustments will also be made for variables for which randomisation did not achieve reasonable balance between arms at baseline, or those associated with missing outcome data.

5.1.2 *Analysis of secondary outcomes over 12 months*

The analysis of secondary outcomes will use similar methods to those for the primary outcomes for continuous variables. For the binary outcomes (remission, sustained remission), the intervention effect will be reported as the odds ratio. Generalized (linear or logistic) random-effects regression models will be used, adjusting for baseline outcome score and clustering, and other baseline variables as above. For outcomes to be examined over the 12 month follow-up period (other than the remission variables), regression models will include a variable to represent 'time' to indicate whether the data was collected at the 6 week, 12 week or 12 month time points. To assess whether the intervention effect varies over time, an intervention x time interaction term will be fitted to allow for a different intervention effect at 6 weeks, 12 weeks, and 12 months, although this will not be highly powered.

5.2 Statistical considerations

Adjustment for multiple outcomes and reporting p-values
No p-value adjustment will be conducted. Interpretation of the intervention effect will be based on the strength of evidence of effect size and consistency of results for related outcomes.

Missing baseline and outcome data
The number (%) of participants with complete data will be reported. If scales have recommended methods for dealing with missing data, these will be applied. As outlined above, primary analyses will be complete case, with adjustments made for variables associated with missingness, to account for missing data. If necessary, in sensitivity analyses, we will apply appropriate methods to impute missing outcome data (see below).

Model assumption checks
For continuous outcomes, model residuals will also be plotted to check for normality and inspected for outliers. If substantial departures from normality occur, transformations will be considered. If a suitable transformation cannot be found, a non-parametric analysis will be considered.

5.3 Planned sub-group (moderator) analyses

A moderator analysis will be conducted to investigate for whom, and under what circumstances, the problem-solving intervention is effective. We will assess modification of intervention effect by *a-priori* defined modifiers (i.e. chronicity of mental health difficulties (<=12 months, > 12 months), severity of mental health difficulties (borderline, abnormal), YTP type (symptomatic, social, both), and SDQ caseness profile (internalizing; externalizing; both internalizing and externalizing; neither), by fitting appropriate interaction terms and testing for heterogeneity of intervention effects in regression models.

5.4 Mediation analyses

A mediation analysis will be conducted to examine whether the following theoretically-driven a priori factors mediate the effects of the intervention on mental health symptoms and idiographic problems at 12 months: perceived stress at 6 weeks, frequency of POD booklet use in the past year , frequency of use of techniques learnt in the past year. All analyses will control for potential confounders including baseline primary outcome and mediator scores (where available) following the approaches used for the main trial analyses. Using generalized structural equation models with bootstrapped confidence intervals, and the causal steps outlined by Baron and Kenny [4], we will examine associations between the intervention and each potential mediator (path a), the mediator and the outcomes (path b), and the intervention and the outcomes (path c). Evidence of an indirect effect (a x b) indicates that mediation is present. The proportion of the intervention effect that is mediated by the mediator is: indirect effect (a x b)/ direct effect (c) * 100.

Figure 2: Path model for mediation

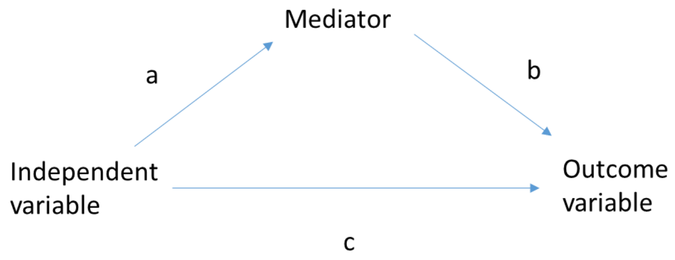

5.5 Adherence analysis

Intervention completion for those in the PRIDE arm is defined as participation in at least 4 PRIDE sessions. We will summarise the intervention received and investigate whether there is a dose effect of the intervention (1-3 PRIDE sessions vs 4 + session) using generalized linear random-effects regression models, adjusting for baseline outcome score and clustering, and other baseline variables as above.

5.6 Additional analyses

Additional secondary analyses will be conducted to answer exploratory questions related to potential intervention mechanisms where data is available.

6. References

1. Parikh R, Michelson D, Malik K, et al. The effectiveness of a low-intensity problem-solving intervention for common adolescent mental health problems in New Dehli, India: protocol for a school-based, individually randomized controlled treatment trial with an embedded stepped-wedge cluster randomized controlled recruitment trial. Trials under review.
2. Schulz KF, Altman DG, Moher D. CONSORT 2010 statement: updated guidelines for reporting parallel group randomised trials. Bmj 2010;340:c332.
3. Sullivan TR, White IR, Salter AB, Ryan P, Lee KJ. Should multiple imputation be the method of choice for handling missing data in randomized trials? Statistical methods in medical research 2018;27:2610-26.
4. Baron RM, Kenny DA. The moderator-mediator variable distinction in social psychological research: conceptual, strategic, and statistical considerations. J Pers Soc Psychol 1986;51:1173-82.

7. Appendix I: Dummy tables

Table A1 Baseline characteristics of completers of outcome evaluation and participants lost to follow-up at 12-month time point (LTFU)

	Lost before 12 months evaluation* (n=)	Completed 12 month outcome evaluation (n=)	p-value [1]
**Gender (n [%])**<br>Female<br>Male			
**Age (years)** (mean [SD])			
**Class (n [%])**<br>9th Class<br>10th Class<br>11th Class<br>12th Class			
**School (n [%])**<br>GBSSS, Mahipalpur.<br>GBSSS, Badarpur.<br>SBV, Badarpur.<br>GGSSS, Badarpur.<br>ASMS-SKV, Mahipalpur.<br>SarvodayaV Co-Ed, Vasant Vihar.			
**Week of enrolment (n [%])**			
**Primary Caregiver (n [%])**<br>Mother & Father<br>Mother<br>Father<br>Grandmother<br>Aunt/Uncle			
**Primary caregiver age (years)** (mean [SD])			
**Primary caregiver gender** (n [%])			
**Caregiver Education**<br>No formal education<br>Completed primary<br>Completed secondary school and above<br>Data Not Available			

**Caregiver Occupation**<br>Unemployed/ Homemaker<br>Unskilled manual work<br>Skilled manual work<br>Professional<br>Data Not Available			
**SDQ Total Difficultly Score** (mean [SD])			
**SDQ Impact score** (mean [SD])			
**SDQ Internalising subscale** (mean [SD])			
**SDQ Externalising subscale** (mean [SD])			
**SDQ prosocial subscale** (mean [SD])			
**SDQ Chronicity (n [%])**<br>1-5 Months<br>6-12 Months<br>Over a Year			
**YTP severity score** (mean [SD])			
**PSS-4 score**			
**SWEMWBS score**			
**Caregiver reported SDQ Total Difficulty score (mean [SD])**			
**Caregiver reported SDQ Impact score (mean [SD])**			
**Caregiver reported SDQ internalising subscale (mean [SD])**			
**Caregiver reported SDQ externalising subscale (mean [SD])**			

*Were deemed eligible, underwent baseline assessment but for whom 6 week outcomes are not available
[1] By Mann-Whitney test where medians are reported, by t-test where means are reported, and by chi2 test for categorical variables.

Table A2. Primary outcomes according to baseline characteristics in the intervention arm

	n	SDQ total score at 12 months weeks (mean [SD])	YTP mean score at 12 months (mean [SD])
**Age group** (years)<br><15yrs<br>15-16<br>17+			
**Gender**<br>Male<br>Female			
**SDQ Total Difficulty Score at baseline**<br>19-20<br>21-23<br>24-25<br>26+			
**SDQ Impact score at baseline**<br>2-3<br>4-5<br>6-7<br>8-10			
**Mean YTP severity score at baseline**<br>0.0-6.0<br>6.1-7.5<br>7.6-9.0<br>9.1-10.0			
**PSS-4 score**<br>2-7<br>8-9<br>10-11<br>12-15			
**SWEMWBS score**<br>10-17<br>18-20<br>21-24<br>25-34			

Table A3: Intervention effect on SDQ total and YTP mean scores at 12 months

	Arm A (EUC)	Arm B (EUC+PRIDE)	Intervention effect		
			Adjusted mean difference (PRIMARY)	Adjusted Effect size (95%CI)[1]	p-value
**SDQ total score (mean [SD])**					
12 months					
**YTP mean score (median [IQR])**					
12 months					

1 Assumes equal standard deviation per arm.

Figure 3. Mean SDQ total difficulties score over time according to arm

Figure 4. Mean YTP mean score over time according to arm

Table A4. Primary Outcome by potential effect modifiers: SDQ total difficulties scores at 12 months

	PRIDE (mean [SD])	EUC (mean [SD])	Intervention effect: adjusted mean difference* [95% CI]	P value for effect modification
**YTP type**				
Symptomatic				
Social				
Both				
**SDQ caseness**				
Elevated internalising				
Elevated externalising				
Both internalising and externalising				
Neither				
**Chronicity of mental health difficulties (SDQ Impact score)**				
<= 12 months				
>12 months				
**Baseline severity of mental health difficulties (SDQ Total difficulties score)**				
Borderline				
Abnormal				
*Adjusted as for the primary analyses (see main text)				

Table A5. Primary Outcome by potential effect modifiers: YTP scores at 12 months

	**PRIDE (mean [SD])**	**EUC (mean [SD])**	**Intervention effect: adjusted mean difference* [95% CI]**	**P value for effect modification**
**YTP type**				
Symptomatic				
Social				
Both				
**SDQ caseness**				
Elevated internalising				
Elevated externalising				
Both internalising and externalising				
Neither				
**Chronicity of mental health difficulties (SDQ Impact score)**				
<= 12 months				
>12 months				
**Baseline severity of mental health difficulties (SDQ Total difficulties score)**				
Borderline				
Abnormal				
*Adjusted as for the primary analyses (see main text)				

Table A6 Dose-response relationship

	SDQ total score at baseline (mean [SD])	YTP mean score at baseline (mean [SD])	SDQ total score at 12 months (mean [SD])[1, 2]	YTP mean score at 12 months (mean [SD]) [1, 2]
**Intervention compliance**<br><br>Non-compliers (0-3 sessions) (n=x, x%)<br>Compliers (4+ session) (n=x, x%)				
**Number of sessions**				
0-1 sessions (n=x)				
2-3 sessions (n=x)				
4-5 sessions (n=x)				

Table A7. Process indicators for trial participants in the PRIDE and EUC arms

	PRIDE			EUC		
	**n**	**SDQ total score at 12 months (mean [SD])**	**YTP mean score at 12 months (mean [SD])**	**n**	**SDQ total score at 12 months (mean [SD])**	**YTP mean score at 12 months (mean [SD])**
**Use of booklets in past 12 months**<br>Never<br>At least once every month<br>At least once every week<br>Daily<br>Others, please specify						
**Use of techniques in past 12 months**<br>Never<br>At least once every month<br>At least once every week<br>Daily<br>Others, please specify						
**Helpfulness of techniques/ lessons learnt over past 12 months**<br>Extremely helpful<br>Very helpful<br>Slightly helpful<br>Not at all helpful						

Table A8: Mediation effect of Perceived stress measured at 6 weeks

**Effect**	**Estimate**	**SE**	**p-value**	**95%Bootstrap**
**SDQ Total difficulties score**				
(c) Intervention → SDQ Total score (12 months)				
(a) Intervention arm → PSS-4 score (6 weeks)				
(b) PSS-4 score (6 weeks) → SDQ Total score (12 months)				
Indirect effect: a x b				
**YTP score**				
(c) Intervention → YTP score (12 months)				
(a) Intervention arm → PSS-4 score (6 weeks)				
(b) PSS-4 score (6 weeks) → YTP score (12 months)				
Indirect effect : a x b				

Table A9: Mediation effect of frequency of POD booklet use in the past 12 months

Effect	Estimate	SE	p-value	95%Bootstrap
**SDQ Total difficulties score**				
(c) Intervention → SDQ Total score (12 months)				
(a) Intervention arm → Frequency POD booklet use (past year)				
(b) Frequency POD booklet use (past year) → SDQ Total score (12 months)				
Indirect effect: a x b				
**YTP score**				
(c) Intervention → YTP score (12 months)				
(a) Intervention arm → Frequency POD booklet use (past year)				
(b) Frequency POD booklet use (past year) → YTP score (12 months)				
Indirect effect : a x b				

Table A10: Mediation effect of frequency of use of techniques learnt in the past 12 months

Effect	Estimate	SE	p-value	95%Bootstrap
**SDQ Total difficulties score**				
(c) Intervention → SDQ Total score (12 months)				
(a) Intervention arm → Frequency use of techniques learnt (past year)				
(b) Frequency use of techniques learnt (past year) → SDQ Total score (12 months)				
Indirect effect: a x b				
**YTP score**				
(c) Intervention → YTP score (12 months)				
(a) Intervention arm → Frequency use of techniques learnt (past year)				
(b) Frequency use of techniques learnt (past year) → YTP score (12 months)				
Indirect effect : a x b				

Table A11. Secondary outcomes: Intervention effect over 12 months[1]

	At 6 weeks		At 12 weeks		At 12 months		Over 12 months				
**Outcome**	**PRIDE arm (n=X)**	**EUC arm (n=X)**	**PRIDE arm (n=X)**	**EUC arm (n=X)**	**PRIDE arm (n=X)**	**EUC arm (n=X)**	**PRIDE arm (n=X)**	**EUC arm (n=X)**	**Adjusted mean difference or odds ratio (95% CI)**	**Adjusted effect size**	**p-value**
**Secondary outcomes**											
Mean self-reported SDQ Total difficulties score over 12 months (SD)											
Mean self-reported YTP severity score over 12 months (SD)											
Mean self-reported SDQ Impact score over 12 months (SD)											
Mean self-reported SDQ internalising subscale score over 12 months (SD)											

[1] This table assumes no effect modification by time – if there is effect modification, results will be shown separately at 6 weeks, 12 weeks, and 12 months.

Mean self-reported SDQ externalising subscale score over 12 months (SD)											
Mean self-reported Perceived Stress Scale-4-item version (PSS-4) total score over 12 months (SD)											
Mean self-reported SWEMWBS score over 12 months (SD)											
Proportion with remission based on self-reported SDQ Total difficulties score and SDQ impact score at 12 months (%)											
Proportion with sustained remission based on self-reported SDQ Total difficulties score and SDQ impact score at 6 weeks, 12 weeks & 12 months (%)											
**Exploratory outcomes**											
Academic outcome 1											
Academic outcome 2											
