# Supplementary material for: Effectiveness and costs associated with a lay counselor–delivered, brief problem-solving mental health intervention for adolescents in urban, low-income schools in India: 12-month outcomes of a randomized controlled trial
Source: PLoS Med. 2021 Sep 28;18(9):e1003778. doi: 10.1371/journal.pmed.1003778 (PMC8478208; doi:10.1371/journal.pmed.1003778)
Supplement: S1 Checklist — CONSORT, Consolidated Standards of Reporting Trials. (DOC) [file pmed.1003778.s002.doc]

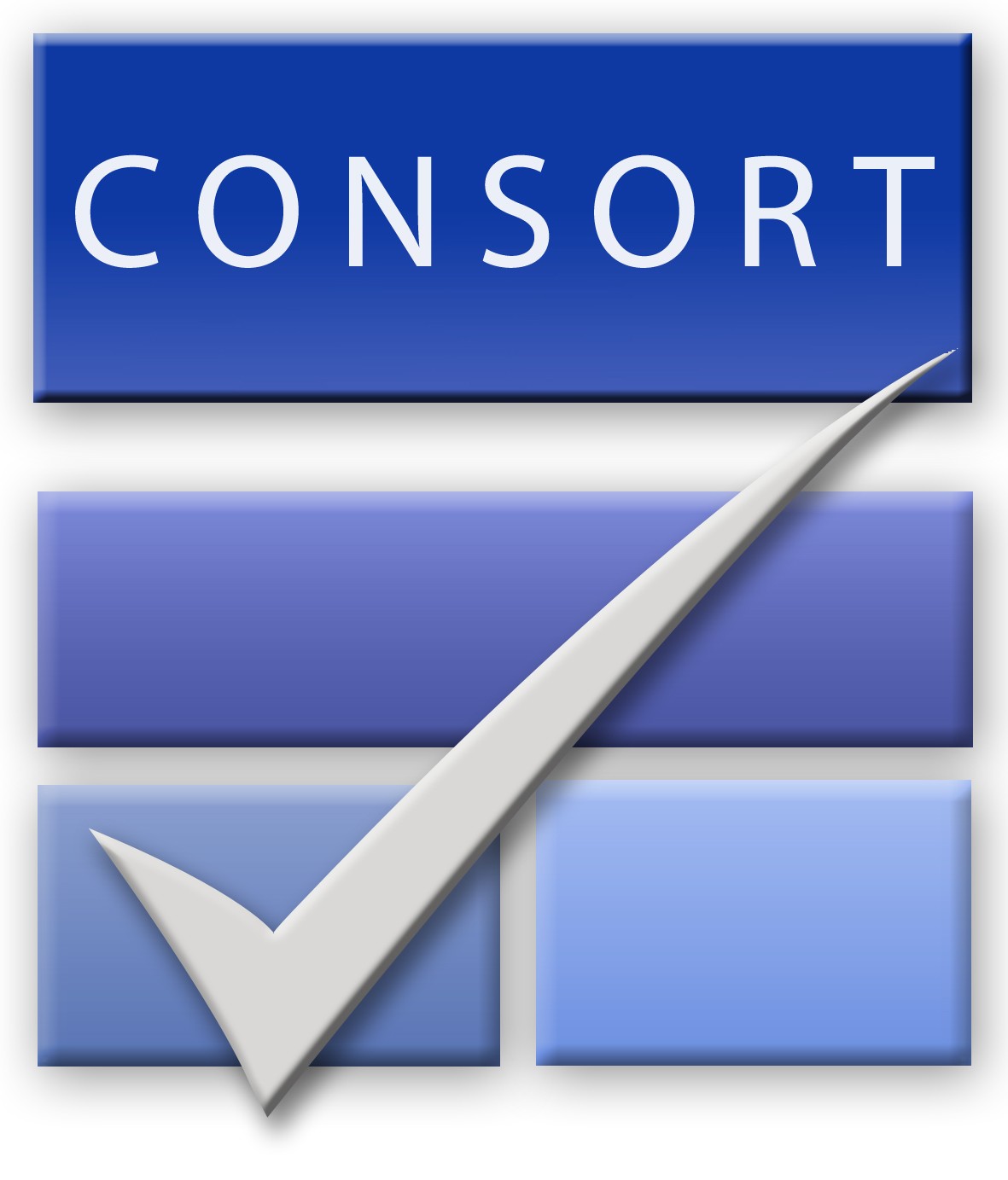
S1 Checklist: CONSORT checklist*

| Section/Topic | Item No | Checklist item | Reported on page No |
| --- | --- | --- | --- |
| Title and abstract | | | |
|  | 1a | Identification as a randomised trial in the title | Title |
|  | 1b | Structured summary of trial design, methods, results, and conclusions (for specific guidance see CONSORT for abstracts) | Abstract |
| Introduction Page | | | |
| Background and objectives | 2a | Scientific background and explanation of rationale | Introduction section, paragraph 1 and 2 |
|  | 2b | Specific objectives or hypotheses | Introduction section, paragraph 2 |
| Methods | | | |
| Trial design | 3a | Description of trial design (such as parallel, factorial) including allocation ratio | Methods- Study design and participants, paragraph 1-3 |
|  | 3b | Important changes to methods after trial commencement (such as eligibility criteria), with reasons | Introduction section, paragraph 2 |
| Participants | 4a | Eligibility criteria for participants | Methods section- Study design and participants sub-section, paragraph 2 |
|  | 4b | Settings and locations where the data were collected | Methods section- Study design and participants sub-section, paragraph 3 |
| Interventions | 5 | The interventions for each group with sufficient details to allow replication, including how and when they were actually administered | Methods section- Intervention and control arms sub-section, paragraph 1-4 |
| Outcomes | 6a | Completely defined pre-specified primary and secondary outcome measures, including how and when they were assessed | Methods section- Outcome measures subsection; Costing framework sub-section; and Study design and participants sub-section, paragraph 3 |
|  | 6b | Any changes to trial outcomes after the trial commenced, with reasons | NA |
| Sample size | 7a | How sample size was determined | Methods section- Study design and participants sub-section, paragraph 3 |
|  | 7b | When applicable, explanation of any interim analyses and stopping guidelines | NA |
| Randomisation: |  |  |  |
| Sequence generation | 8a | Method used to generate the random allocation sequence | Methods section- Study design and participants sub-section, paragraph 3 |
|  | 8b | Type of randomisation; details of any restriction (such as blocking and block size) | Methods section- Study design and participants sub-section, paragraph 3 |
| Allocation concealment mechanism | 9 | Mechanism used to implement the random allocation sequence (such as sequentially numbered containers), describing any steps taken to conceal the sequence until interventions were assigned | Methods section- Study design and participants sub-section, paragraph 3 |
| Implementation | 10 | Who generated the random allocation sequence, who enrolled participants, and who assigned participants to interventions | Methods section- Study design and participants sub-section, paragraph 3 |
| Blinding | 11a | If done, who was blinded after assignment to interventions (for example, participants, care providers, those assessing outcomes) and how | NA as these were self-reported assessments |
|  | 11b | If relevant, description of the similarity of interventions | NA |
| Statistical methods | 12a | Statistical methods used to compare groups for primary and secondary outcomes | Statistical analsysis section, Effectiveness analysis subsection, paragraph 1 |
|  | 12b | Methods for additional analyses, such as subgroup analyses and adjusted analyses | Statistical analsysis section, Effectiveness analysis subsection, and Cost Analysis section |
| Results | | | |
| Participant flow (a diagram is strongly recommended) | 13a | For each group, the numbers of participants who were randomly assigned, received intended treatment, and were analysed for the primary outcome | Figure 1. Table 2 |
|  | 13b | For each group, losses and exclusions after randomisation, together with reasons | Figure 1 |
| Recruitment | 14a | Dates defining the periods of recruitment and follow-up | Methods section- Study design and participants sub-section, paragraph 1 and 3 |
|  | 14b | Why the trial ended or was stopped | Methods section- Study design and participants sub-section, paragraph 3 |
| Baseline data | 15 | A table showing baseline demographic and clinical characteristics for each group | Table 1 |
| Numbers analysed | 16 | For each group, number of participants (denominator) included in each analysis and whether the analysis was by original assigned groups | Table 2 |
| Outcomes and estimation | 17a | For each primary and secondary outcome, results for each group, and the estimated effect size and its precision (such as 95% confidence interval) | Table 2; Results section, Effectiveness analysis sub-section |
|  | 17b | For binary outcomes, presentation of both absolute and relative effect sizes is recommended | Table 2, Results section, Effectiveness analysis sub-section, paragraph 2 |
| Ancillary analyses | 18 | Results of any other analyses performed, including subgroup analyses and adjusted analyses, distinguishing pre-specified from exploratory | Results section, Moderator, mediators and process indicators subsection; and Incremental cost and modelled scale-up cost sub-section |
| Harms | 19 | All important harms or unintended effects in each group (for specific guidance see CONSORT for harms) | Results section, paragraph 1 |
| Discussion | | | |
| Limitations | 20 | Trial limitations, addressing sources of potential bias, imprecision, and, if relevant, multiplicity of analyses | Discussion section, paragraph 5 |
| Generalisability | 21 | Generalisability (external validity, applicability) of the trial findings | Discussion section, paragraph 6 |
| Interpretation | 22 | Interpretation consistent with results, balancing benefits and harms, and considering other relevant evidence | Discussion section, paragraph 1-4 |
| Other information | | |  |
| Registration | 23 | Registration number and name of trial registry | ClinicalTrials.gov; NCT03630471 |
| Protocol | 24 | Where the full trial protocol can be accessed, if available | Supplementary file, S1 Appendix |
| Funding | 25 | Sources of funding and other support (such as supply of drugs), role of funders | Financial Disclosure statement |

*We strongly recommend reading this statement in conjunction with the CONSORT 2010 Explanation and Elaboration for important clarifications on all the items. If relevant, we also recommend reading CONSORT extensions for cluster randomised trials, non-inferiority and equivalence trials, non-pharmacological treatments, herbal interventions, and pragmatic trials. Additional extensions are forthcoming: for those and for up to date references relevant to this checklist, see [www.consort-statement.org](http://www.consort-statement.org/).
