# Supplementary material for: Effectiveness and costs associated with a lay counselor–delivered, brief problem-solving mental health intervention for adolescents in urban, low-income schools in India: 12-month outcomes of a randomized controlled trial
Source: PLoS Med. 2021 Sep 28;18(9):e1003778. doi: 10.1371/journal.pmed.1003778 (PMC8478208; doi:10.1371/journal.pmed.1003778)
Supplement: S1 Text — (DOCX) [file pmed.1003778.s003.docx]

**S1 Text: Assumptions guiding cost estimates for the modelled scale-up of the counsellor-led problem-solving intervention in 20 schools for one academic year**

We prepared the scale-up costs by assuming that there would be 20 government-run schools in a block, one counsellor per school, and three supervisors, each supervising 6-7 counsellors. The schools were assumed to be accessible for six working hours per day and six working days per week. The counsellors were assumed to be employed full-time on fixed-term 9-month contracts (39 weeks), which does not include the end of term and summer vacation period as per the convention for employing associate staff in schools of Delhi. The implementing partner organization (Sangath) was assumed to employ the supervisors and their time was charged as a fraction of full-time salary. It was also assumed that providers would be school counsellors with no previous training in evidence-based interventions and that supervisors would be psychologists with 2-3 years of experience in delivering evidence-based interventions.

In terms of the work scope, it was assumed that each counsellor would engage exclusively in activities related to the problem-solving intervention including screening, referral generation, intervention delivery, associated training and supervision. Based on time allocation in the main trial, the division of time for each of these activities over 39 weeks was prepared (Table A). We took into account that no intervention would be possible for seven weeks of the academic year due to exams and holidays (even though counsellors would be paid during this time), leaving 32 weeks for activities directly related to PRIDE training and delivery. A total of 8 weeks was set aside for office and field-based training. In the remaining 24 weeks, with four days per week available for intervention delivery (excluding time for screening, sensitization and supervision) and assuming a single session duration of 0.67 hours (including actual session time, time for writing notes and accompanying adolescent to/from the classroom before/after the session), a maximum of 768 sessions were estimated as being possible in an academic year (8 sessions per day x 4 days per week x 24 weeks). The original trial [1] found that the average session attendance was 4.06 for each participant. Extrapolating from this figure, the intervention could be delivered to 189 adolescents per school, providing coverage to 18% of the school population in the targeted grades (1060 being average size of the targeted student cohort in each school).

The itemized list of human resources, materials and supplies are provided in Table A and B. All the items listed in the table were included in the cost estimates provided in the main text. In estimating the costs for scale-up, we assumed each school would have its own infrastructure (chairs, tables, space for storage, projector and laptops), so these were not included in the cost analysis. Printable intervention materials have been developed by the project team and are intended for distribution free of charge. Thus, future development costs to modify these materials would be marginal and are therefore not included in this analysis. However, the cost of printing materials and additional supplies required for delivering the intervention have been included in cost estimates.

**Table A: Division of counsellor and supervisor time for intervention scale-up across 20 schools for one academic year**

| **Activity** | **Frequency** | **Counsellor time^[[1]](#footnote-1)^ (multiplied by number of counsellors involved)** | **Supervisor time ^[[2]](#footnote-2)^ (multiplied by number supervisors involved)** |
| --- | --- | --- | --- |
| Office-based training | 5 days | 5 days (x 20) | 30 hours (x 3) |
| Field-based training – sessions | 6 weeks | 36 days (x 20) | - |
| Field-based training – group supervision | Once per week up to 6 weeks | 0.6 day/week for 6 weeks (x 20) (includes travel time) | 2.5 hours/week for 6 weeks (x 3) |
| Field-based training – individual supervision | Once per week up to 6 weeks | 0.13 day/week for 6 weeks (x 20) | 5 hours/week for 6 weeks (x 3) |
| Field visit | 1 visit per week for 6 weeks |  | 4 hours per week (x3) for 6 weeks (includes travel time between school and office) |
| Refresher training | 4 days | 4 days (x 20) | 24 hours (x 3) |
| Group supervision | Once per week for 24 weeks | 0.6 day/week for 24 weeks (x 20) (include travel time between school and office) | 2.5 hours/week for 24 weeks (x 3) |
| Supervision calls | Once per week for 24 weeks | 0.04 day/week for 24 weeks (x 20) | 2 hours/week (x3) + 1 hour per week for documentation, extended supervision for cases with serious adverse events. |
| Screening cases | 1 day per week for 24 weeks | 1 day /week for 24 weeks (x 20) | - |
| Sensitization and referral generation | 30 classroom sessions and follow-ups | 0.36 day /week for 24 weeks (x 20) | - |
| Problem solving intervention | 4 days per week for 24 weeks | 4 days/week for 24 weeks (x 20) | - |

**Table B: Materials and supplies required for intervention scale-up across 20 schools for one academic year**

| **Materials and supplies** | **No. of units^[[3]](#footnote-3)^** |
| --- | --- |
| Travelling allowance for supervisor | 1 school visit per year x 20 schools |
| Phone allowance | Monthly x 20 counsellors |
| Laptops^[[4]](#footnote-4)^ | 1 per supervisor x 3 supervisors |
| Manuals, formulation sheet, treatment ladder | 1 each per counsellor x 20 counsellors |
| Drop-box | 1 per school x 20 schools |
| Resource material (booklets, poster, monitoring tool, case record form, safety sheet) | 1 per case x 189 cases per school x 20 schools |
| Voice recorders | 1 per counsellor x 20 counsellors |
| Sensitization poster | 5 per school x 20 schools |
| Hospital screens for creating private space | 1 per school x 20 schools |

**Reference:**

1. Michelson D, Malik K, Parikh R, Weiss HA, Doyle AM, Bhat B, et al. Effectiveness of a brief lay counsellor-delivered, problem-solving intervention for adolescent mental health problems in urban, low-income schools in India: a randomised controlled trial. Lancet Child Adolesc Health. 2020 Aug;4(8):571–82.

1. Average monthly salary of the school counsellor in government-run school of Delhi: INR 26850. [↑](#footnote-ref-1)
2. Average monthly salary of supervisor is INR 63600 as per the technical organization scale (source Sangath). Costs were prorated from per hour cost [↑](#footnote-ref-2)
3. The supplies were costed as per the actual expenditure during trial. [↑](#footnote-ref-3)
4. The cost of laptops was adjusted as per the time involvement of supervisor in the project. [↑](#footnote-ref-4)
