# Supplementary material for: Effectiveness and costs associated with a lay counselor–delivered, brief problem-solving mental health intervention for adolescents in urban, low-income schools in India: 12-month outcomes of a randomized controlled trial
Source: PLoS Med. 2021 Sep 28;18(9):e1003778. doi: 10.1371/journal.pmed.1003778 (PMC8478208; doi:10.1371/journal.pmed.1003778)
Supplement: S1 Table — (DOCX) [file pmed.1003778.s004.docx]

### **S1 Table: Baseline characteristics of participants who completed follow-up (n=174) and those lost to follow-up (n=76) at 12 months^[[1]](#footnote-1)^**

|  | **Lost to 12 months follow-up (n=74)** | **Completed 12 months follow-up (n=176)** | **p-value^[[2]](#footnote-2)^** |
| --- | --- | --- | --- |
| **Age** (in years), mean (SD) | 15.77 (1.67) | 15.55 (1.68) | 0.33 |
| **Sex,** n (%)  Females (n=76)  Males (n=174) | 22 (28.9%)  52 (29.9%) | 54 (71.1%)  122 (70.1%) | 0.88 |
| **School grade**, n (%)  9^th^ Class (n=110)  10^th^ Class (n=67)  11^th^ Class (n=16)  12^th^ Class (n=57) | 28 (25.5%)  18 (26.9%)  3 (18.8%)  25 (43.9%) | 82 (74.5%)  49 (73.1%)  13 (81.3%)  32 (56.1%) | 0.06 |
| **Primary caregiver age** (in years), mean (SD) | 35.79 (9.47) | 37.31 (8.21) | 0.22 |
| **Primary caregiver education^[[3]](#footnote-3)^,** n (%)  No formal education (n=50)  Completed primary (n=5)  Completed secondary school (n=113)  Completed higher-secondary and above (n=51)  Data not available (n=17) | 15 (30.0%)  1 (20.0%)  28 (24.8%)  18 (35.3%)  4 (23.5%) | 35 (70.0%)  4 (80.0%)  85 (75.2%)  33 (64.7%)  13 (76.5%) | 0.79 |
| **Primary caregiver occupation**^3^**,** n (%)  Not employed outside home (n=91)  Manual (n=111)  Clerical (n=14)  Professional (n=7)  Other (n=13) | 25 (27.5%)  36 (32.4%)  2 (14.3%)  2 (28.6%)  1 (7.7%) | 66 (72.5%)  75 (67.6%)  12 (85.7%)  5 (71.4%)  12 (92.3%) | 0.51 |
| **SDQ Total Difficulties Score^[[4]](#footnote-4)^,** mean (SD) | 22.76 (2.88) | 23.35 (3.26) | 0.18 |
| **SDQ Impact score**^4^**,** mean (SD) | 4.97 (2.51) | 5.43 (2.32) | 0.17 |
| **SDQ Internalizing subscale**^4^**,** mean (SD) | 11.82 (2.36) | 12.11 (2.51) | 0.41 |
| **SDQ Externalizing subscale^4^,** mean (SD) | 10.93 (2.12) | 11.24 (2.48) | 0.35 |
| **SDQ Chronicity**^4^, n (%)  1-5 Months (n=76)  6-12 Months (n=39)  Over a Year (n=134) | 18 (23.7%)  13 (33.3%)  43 (32.1%) | 58 (76.3%)  26 (66.6%)  91 (67.9%) | 0.38 |
| **YTP score,** mean (SD) | 7.74 (1.94) | 7.11 (2.20) | 0.03 |
| **PSS-4 score,** mean (SD) | 9.01 (2.22) | 9.18 (2.58) | 0.64 |
| **SWEMWBS score,** mean (SD) | 19.96 (4.76) | 21.02 (5.15) | 0.13 |

SDQ=Strengths and Difficulties Questionnaire. YTP=Youth Top Problems measure. PSS-4=Perceived Stress Scale 4-item version. SWEMWBS=Short Warwick-Edinburgh Mental Well-Being Scale.

1. Denominator for each cell is row total [↑](#footnote-ref-1)
2. p-value by t-test where means are reported, and by chi-square test for categorical variables. [↑](#footnote-ref-2)
3. Primary caregiver data not collected for 14 participants as they were above 18 years. [↑](#footnote-ref-3)
4. Baseline SDQ was missing for one participant, see Michelson et al. [7] [↑](#footnote-ref-4)
