# Supplementary material for: Effectiveness and costs associated with a lay counselor–delivered, brief problem-solving mental health intervention for adolescents in urban, low-income schools in India: 12-month outcomes of a randomized controlled trial
Source: PLoS Med. 2021 Sep 28;18(9):e1003778. doi: 10.1371/journal.pmed.1003778 (PMC8478208; doi:10.1371/journal.pmed.1003778)
Supplement: S2 Table — (DOCX) [file pmed.1003778.s005.docx]

**S2 Table: Sensitivity analysis using imputed data for primary and secondary outcomes**

|  | **Sensitivity analsysis adjusted for missing data** | **Sensitivity analsysis adjusting for 16 cases that received additional intervention** |
| --- | --- | --- |
| **Outcome** | **Adjusted mean difference or odd ratio (95%CI); P-value** | **Adjusted mean difference or odd ratio (95%CI); P-value** |
| SDQ Total Difficulties score | At 12 months: -1.22 (-2.96, 0.51); p=0.17 | At 12 months: -1.58 (-3.39, 0.22); p=0.085 |
|  | Over 12 months: -1.02 (-2.13, 0.10); p=0.07 | Over 12 months: -1.35 (-2.54, -0.15); p=0.03 |
| YTP score | At 12 months: -0.72 (-1.39, -0.06); p=0.03 | At 12 months: -0.85 (-1.44, -0.25); p=0.005^[[1]](#footnote-1)^ |
|  | Over12 months: -0.91(-1.41, -0.41); p<0.001 | Over12 months: -1.09 (-1.32, -0.86); p<0.00^1^ |
| SDQ Impact score | Over 12 months: -0.42 (-1.73, 0.90); p=0.54 | Over 12 months: -0.59 (-1.00, 0.17); p=0.006 ^1^ |
| SDQ internalizing subscale | Over 12 months: -0.64 (-1.28, -0.02); p=0.05 | Over 12 months: -0.78 (-1.46, -0.09); p=0.03 |
| SDQ externalizing subscale score | Over12 months: -0.42 (-1.01, 0.17); p=0.16 | Over12 months: -0.57 (-1.21, 0.07); p=0.08 |
| PSS-4 total score | Over12 months: -0.49 (-0.94, -0.04); p=0.03 | Over12 months: -0.63 (-1.10, -0.15); p=0.01 |
| SWEMWBS score | Over12 months: 1.11 (-0.06, 2.28); p=0.06 | Over12 months: 1.24 (0.77, 1.72); p<0.001^1^ |
| Proportion with remission (%) | At 12 months: 1.27 (0.71, 2.26); p=0.43 | At 12 months: 1.02 (0.47, 2.19); p=0.97 |

SDQ=Strengths and Difficulties Questionnaire. YTP=Youth Top Problems measure. PSS-4=Perceived Stress Scale 4-item version. SWEMWBS=Short Warwick-Edinburgh Mental Well-Being Scale

1. Estimated using robust standard errors due to heteroskedasticity. [↑](#footnote-ref-1)
