# Supplementary material for: Effectiveness and costs associated with a lay counselor–delivered, brief problem-solving mental health intervention for adolescents in urban, low-income schools in India: 12-month outcomes of a randomized controlled trial
Source: PLoS Med. 2021 Sep 28;18(9):e1003778. doi: 10.1371/journal.pmed.1003778 (PMC8478208; doi:10.1371/journal.pmed.1003778)
Supplement: S3 Table — SDQ, Strengths and Difficulties Questionnaire. (DOCX) [file pmed.1003778.s006.docx]

**S3 Table: Primary outcome (SDQ) by potential effect modifiers at 12 months**

|  | **Control arm**  **mean (SD)** | **Intervention arm**  **mean (SD)** | | **Intervention effect, adjusted mean difference^[[1]](#footnote-1)^ (95%CI)** | | **p-value for effect modification** |
| --- | --- | --- | --- | --- | --- | --- |
| **YTP typology** | | | | | |  |
| Syndromic | 15.77 (5.40) | | 10.94 (6.69) | | -5.07 (-9.43, -0.70) | 0.21 |
| Functional | 13.34 (5.69) | | 14.23 (6.48) | | -0.24 (-3.38, 2.90) |  |
| Both | 14.17 (6.39) | | 13.13 (5.43) | | -1.50 (-3.94, 0.94) |  |
| **Baseline chronicity of mental health difficulties** | | | | | |  |
| <= 12 months | 13.90 (6.19) | | 13.50 (6.18) | | -1.08 (-3.65, 1.49) | 0.51 |
| >12 months | 14.65 (5.71) | | 12.69 (6.03) | | -2.26 (-4.68, 0.17) |  |
| **Baseline severity of mental health difficulties** | | | | | |  |
| Borderline | 13.83 (3.71) | | 9.96 (5.47) | | -4.27 (-7.62, -0.92)  (p=0.012) | 0.06 |
| Abnormal | 14.41 (6.52) | | 14.28 (5.90) | | -0.53 (-2.55, 1.50) |  |

YTP=Youth Top Problems. SDQ=Strengths and Difficulties Questionnaire

1. Adjusted as for the primary analyses (see main text). [↑](#footnote-ref-1)
