# Supplementary material for: Effectiveness and costs associated with a lay counselor–delivered, brief problem-solving mental health intervention for adolescents in urban, low-income schools in India: 12-month outcomes of a randomized controlled trial
Source: PLoS Med. 2021 Sep 28;18(9):e1003778. doi: 10.1371/journal.pmed.1003778 (PMC8478208; doi:10.1371/journal.pmed.1003778)
Supplement: S4 Table — YTP, Youth Top Problems. (DOCX) [file pmed.1003778.s007.docx]

**S4 Table: Primary outcome (YTP) by potential effect modifiers at 12 months**

|  | **Control arm**  **mean (SD)** | **Intervention arm**  **mean (SD)** | | **Intervention effect, adjusted mean difference^[[1]](#footnote-1)^ (95%CI)** | | **p-value for effect modification** |
| --- | --- | --- | --- | --- | --- | --- |
| **YTP problem typology** | | | | | |  |
| Syndromic | 3.18 (3.44) | | 1.77 (2.41) | | -1.23 (-3.06, 0.59) | 0.80 |
| Functional | 2.68 (2.49) | | 2.74 (2.75) | | -0.47 (-1.78, 0.84) |  |
| Both | 2.98 (2.56) | | 2.06 (1.96) | | -0.78 (-1.80, 0.23) |  |
| **Baseline chronicity of mental health symptoms** | | | | | |  |
| <= 12 months | 2.50 (2.34) | | 1.87 (2.19) | | -0.90 (-1.97, 0.17) | 0.77 |
| >12 months | 3.37 (2.92) | | 2.50 (2.42) | | -0.67 (-1.68, 0.34) |  |
| **Baseline severity of mental health symptoms** | | | | | |  |
| Borderline | 3.53 (2.66) | | 1.64 (1.91) | | -1.79 (-3.20, -0.38) | 0.09 |
| Abnormal | 2.73 (2.65) | | 2.45 (2.45) | | -0.37 (-1.21, 0.48) |  |

YTP=Youth Top Problems

1. Adjusted as for the primary analyses (see main text). [↑](#footnote-ref-1)
