# Supplementary material for: Effectiveness and costs associated with a lay counselor–delivered, brief problem-solving mental health intervention for adolescents in urban, low-income schools in India: 12-month outcomes of a randomized controlled trial
Source: PLoS Med. 2021 Sep 28;18(9):e1003778. doi: 10.1371/journal.pmed.1003778 (PMC8478208; doi:10.1371/journal.pmed.1003778)
Supplement: S5 Table — SDQ, Strengths and Difficulties Questionnaire. (DOCX) [file pmed.1003778.s008.docx]

**S5 Table: Mediation effect of perceived stress, use of problem-solving materials and problem-solving skills on SDQ Total Difficulties score at 12 months**

|  | **Estimate** | **SE** | **p-value** | **95%Bootstrap CI** |
| --- | --- | --- | --- | --- |
| **Mediation effect of perceived stress** | | | | |
| Total effect: Intervention effect on SDQ Total Difficulties score (12 months) | -1.40 | 0.93 | 0.13 | -3.23, 0.43 |
| (a) Intervention effect on PSS-4 score (12 weeks) | -0.31 | 0.33 | 0.34 | -0.97, 0.34 |
| (b) PSS-4 score (12 weeks) effect on SDQ Total Difficulties score (12 months) | 0.77 | 0.18 | <0.001 | 0.42, 1.12 |
| *Indirect effect: a x b* | *-0.24* | *0.28* | *0.38* | *-0.79*, *0.30* |
| **Mediation effect of using problem-solving materials** | | | | |
| Total effect: Intervention effect on SDQ Total Difficulties score (12 months) | -1.34 | 0.93 | 0.15 | -3.15, 0.47 |
| (a) Intervention effect on use of problem-solving materials in past year (i.e., did the participant use problem-solving materials at any point in the past year) | -0.14 | 0.06 | 0.03 | -0.27, -0.01 |
| (b) Use of problem-solving materials (in past year) effect on SDQ Total Difficulties score (12 months) | -0.98 | 1.26 | 0.43 | -3.45, 1.48 |
| *Indirect effect: a x b* | *0.14* | *0.21* | *0.51* | *-0.27*, *0.54* |
| **Mediation effect of using problem-solving skills** | | | | |
| Total effect: Intervention effect on SDQ Total Difficulties score (12 months) | -1.34 | 0.93 | 0.15 | -3.15, 0.48 |
| (a) Intervention effect on use of problem-solving skills in past year (i.e., did the participant use skills at any point in the past year) | 0.10 | 0.06 | 0.10 | -0.02, 0.22 |
| (b) Use of problem-solving skills (in past year) effect on SDQ Total Difficulties score (12 months) | -1.72 | 1.34 | 0.20 | -4.35, 0.91 |
| *Indirect effect: a x b* | *-0.17* | *0.21* | *0.41* | *-0.58,* *0.24* |

SDQ=Strengths and Difficulties Questionnaire. PSS= Perceived Stress Scale.
