# Supplementary material for: Effectiveness and costs associated with a lay counselor–delivered, brief problem-solving mental health intervention for adolescents in urban, low-income schools in India: 12-month outcomes of a randomized controlled trial
Source: PLoS Med. 2021 Sep 28;18(9):e1003778. doi: 10.1371/journal.pmed.1003778 (PMC8478208; doi:10.1371/journal.pmed.1003778)
Supplement: S6 Table — YTP, Youth Top Problems. (DOCX) [file pmed.1003778.s009.docx]

**S6 Table: Mediation effect of perceived stress, use of problem-solving materials and problem-solving skills on YTP at 12 months**

|  | **Estimate** | **SE** | **p-value** | **95%Bootstrap** |
| --- | --- | --- | --- | --- |
| **Mediation effect of perceived stress** | | | | |
| Total effect: Intervention effect on YTP score (12 months) | -0.73 | 0.37 | .05 | -1.45, 0.0008 |
| (a) Intervention effect on PSS-4 score (12 weeks) | -0.31 | 0.33 | .34 | -0.97, 0.34 |
| (b) PSS-4 score (12 weeks) effect on YTP score (12 months) | 0.23 | 0.07 | <.001 | 0.09, 0.37 |
| *Indirect effect: a x b* | *-0.07* | *0.09* | *.40* | *-0.24, 0.10* |
| **Mediation effect of using problem-solving materials** | | | | |
| Total effect: Intervention effect on YTP score (12 months) | -0.69 | 0.37 | .06 | -1.42, 0.04 |
| (a) Intervention effect on use of problem-solving materials in past year (i.e., did the participant use materials at any point in the past year) | -0.14 | 0.06 | .03 | -0.27, -0.01 |
| (b) Use of problem-solving materials (in past year) effect on YTP score (12 months) | -0.48 | 0.43 | .26 | -1.32, 0.35 |
| *Indirect effect: a x b* | *0.07* | *0.07* | *.35* | *-0.07, 0.21* |
| **Mediation effect of using problem-solving skills** | | | | |
| Total effect: Intervention effect on YTP score (12 months) | -0.69 | 0.37 | .06 | -1.42, 0.04 |
| (a) Intervention arm effect on use of problem-solving skills in past year (i.e., did the participant use skills at any point in the past year) | 0.10 | 0.06 | .10 | -0.02, 0.22 |
| (b) Use of problem-solving skills (in past year) effect on YTP score (12 months) | -0.82 | 0.53 | .12 | -1.85, 0.22 |
| *Indirect effect: a x b* | *-0.08* | *0.09* | *.34* | *-0.25, 0.09* |

YTP=Youth Top Problems. PSS= Perceived Stress Scale.
