# Supplementary material for: Effectiveness and costs associated with a lay counselor–delivered, brief problem-solving mental health intervention for adolescents in urban, low-income schools in India: 12-month outcomes of a randomized controlled trial
Source: PLoS Med. 2021 Sep 28;18(9):e1003778. doi: 10.1371/journal.pmed.1003778 (PMC8478208; doi:10.1371/journal.pmed.1003778)
Supplement: S7 Table — (DOCX) [file pmed.1003778.s010.docx]

**S7 Table: Dose-response effect on primary outcomes at 12 months for intervention completers and non-completers**

|  | **Baseline**  **Mean (SD)** | **12 months**  **Mean (SD)** | **Adjusted mean difference (95%CI)** ^[[1]](#footnote-1)^ | **p-value** |
| --- | --- | --- | --- | --- |
| **SDQ Total Difficulties score** | | | | |
| Non-completers^[[2]](#footnote-2)^ | 23.07 (3.99)  (n=27) | 8.38 (5.85)  (n=13) | 5.20 (1.72, 8.68) | 0.003 |
| Completers^[[3]](#footnote-3)^ | 23.27 (3.12)  (n=98) | 13.94 (5.73)  (n=68) |  |  |
| **YTP score** | | | | |
| Non-completers2 | 7.09 (2.51)  (n=27) | 1.50 (2.20)  (n=13) | 0.91 (-0.48, 2.29) | 0.20 |
| Completers3 | 7.28 (2.17)  (n=98) | 2.36 (2.34)  (n=68) |  |  |

SDQ=Strengths and Difficulties Questionnaire. YTP=Youth Top Problems measure.

1. Coefficients from mixed-effects regression model adjusting for baseline value of outcome, age group, school, class, mean YTP score at baseline and counsellor [↑](#footnote-ref-1)
2. Non-completers were defined as those attending less than 3 sessions [↑](#footnote-ref-2)
3. Completers were defined as those attending 4-5 sessions [↑](#footnote-ref-3)
