# Supplementary material for: Effectiveness and costs associated with a lay counselor–delivered, brief problem-solving mental health intervention for adolescents in urban, low-income schools in India: 12-month outcomes of a randomized controlled trial
Source: PLoS Med. 2021 Sep 28;18(9):e1003778. doi: 10.1371/journal.pmed.1003778 (PMC8478208; doi:10.1371/journal.pmed.1003778)
Supplement: Attachment — Submitted filename: point by point responses.docx [file pmed.1003778.s011.docx]

**Authors’ Responses to editors’ and reviewers’ comments**

Dear Editor,

Thank you for your comments as well as those from the reviewers. We have provided point-by-point responses to your comment. The editors’ and reviewers’ comments are presented in the first column followed by authors’ responses and edits to the manuscript in the second column. The page and line numbers correspond to the cleaned version of the revised manuscript draft.

|  | **Editors/ Reviewers Comments** | **Authors’ responses** |
| --- | --- | --- |
| **Editors’ Comments** | |  |
| 1. | Data availability statement: Please revise the data statement. PLOS Medicine requires that the de-identified data underlying the specific results in a published article be made available, without restrictions on access, in a public repository or as Supporting Information at the time of article publication, provided it is legal and ethical to do so | **The de-identified data can be accessed through https://datacompass.lshtm.ac.uk/** |
| 2. | Competing Interests: Please add this statement to the manuscript's Competing Interests: "VP is an Academic Editor on PLOS Medicine's editorial board." | **The suggested statement has been added to the Competing Interests.** |
| 3. | Abstract: Methods and Findings: At line 47-48, please indicate you are reporting mean age of the participants. Please provide some demographic background on the adolescent participants, beyond age, such as the population and setting, years during which the study took place. | **The description of participant characteristics has been updated in the abstract (page 3, line number 47-50). We have also revised the sample size to account for the one adolescent who withdrew consent after randomization**  Participants in the original trial were 250 school-going adolescents (Mean age=15.61 years, SD=1.68), including 174 (69%) who identified as males. Participants were recruited from six government schools over a period of 4 months (20^th^ August-14^th^December 2018) and were selected on the basis of elevated mental health symptoms and distress/functional impairment. |
| 4. | Abstract: Methods and Findings: The retrospective ingredients approach taken could be mentioned. | **This detail has been added to the abstract (page 4, line number 64), as below:**  Costs were estimated for delivering the intervention during the trial and from modelling a scale-up scenario, using the retrospective ingredients approach. |
| 5. | Abstract: Methods and Findings: For the YTP and SDQ main outcomes, please quantify the main results with both 95% CIs and p values. | **These details have been added to the abstract (page 4, line number 66-69), as below:**  There was evidence for an intervention effect on both primary outcomes at 12 months (YTP: AMD=-0.75; 95%CI=-1.47, -0.03; p=0.04; SDQ Total Difficulties: AMD=-1.73, 95% CI= -3.47, 0.02, p=0.05), with stronger effects over 12 months (YTP: AMD=-0.98; 95%CI=-1.51, -0.45, p<0.001; SDQ: AMD=-1.23, 95%CI=-2.37, -0.09, p=0.03). |
| 6. | Abstract: Conclusions: We suggest beginning the first sentence with “In this study, we observed…” or similar. | **The suggested text insertion has been made to the abstract (page 4, line number 74), as below:**  In this study, we observed that a lay counsellor-delivered, brief transdiagnostic problem-solving intervention had sustained effects on psychosocial problems and mental health symptoms over 12 months follow-up period. |
| 7. | Abstract and Author Summary: Where you mention that effects were “moderated by the pre-intervention severity of mental health problems.” could you be more specific in terms of the moderation so the direction/relationship is clear. | **In light of the editors' comment about the moderation effect not meeting the statistical significance (p=0.06 and p=0.09), we have removed the text describing moderation effect from both abstract and author summary.** |
| 8. | Methods: While described elsewhere (in ref 8) it would be helpful if some details could be briefly mentioned, such as: briefly summary of details of recruitment and randomization, and background on the lay-counsellors, some details of a definition for the “remission” outcome. | **Further details about recruitment have been added to Methods (page 9, line number 156-159), as below:**  Referrals into the trial were generated through whole-school and classroom-level sensitization activities that were intended to (i) raise awareness of available mental health support, and (ii) address factors such as low mental health literacy and confidentiality concerns that might otherwise limit the demand for school mental health services.  **Further details about randomization have been added to Methods (page 10, line number 175-177), as below:**  Participants were randomized using a randomization list generated by the senior trial statistician and concealed using sequentially numbered sealed envelopes [80.  **Further details about the counsellors’ background have been added to Page 11 (line number 209-210), as below:**  The intervention was delivered by eight Hindi-speaking, college graduates who had no formal clinical qualifications or experience of delivering evidence-based psychological treatments. The counsellors were employed by the implementing organization (Sangath) and trained through a combination of office-based learning and supervised field practice. Counsellors also received weekly group supervision; details are described elsewhere [7].  **Further details about remission criteria have been added to Outcome measures section (page 13, line number 253-254), as below:**  … and (vi) proportion of remitted cases (assessed using the ‘crossing clinical threshold method’ [14] and defined as scoring below eligibility thresholds on both the SDQ Total Difficulties score and SDQ Impact score [8]). |
| 9. | Methods: Line 244: Thank you for your note that the statistical analysis plan is available upon request. Please include this as a supporting information file. In addition, you reference the published protocol- however this does not seem to include the updated 12-month time point. It would be helpful if you could please include the modified protocol as a supporting information file. | **As mentioned in the previous manuscript draft, the trial steering committee recommended a post-hoc extension of the follow-up period to 12 months, which was subsequently approved by the two IRBs (Harvard Medical School, USA and Sangath, India). The statistical analysis plan was finalized and signed before undertaking the analysis of the 12-month follow-up results. This analysis plan has been provided as a supporting information file, which also serves as the study protocol for the 12-month follow-up study (S1 Appendix).** |
| 10. | Results: Lines 355-356: Please indicate in the text that the effect on SWEMWBS score did not reach statistical significance. | **The text now specifies that a non-significant effect was observed on the SWEMWBS, as below (Results section, Page 26, line 398-400):**  A modest effect was observed on the SWEMWBS score and this did not reach statistical significance (AMD=1.16, 95% CI=-0.07, 2.38; d=0.19, 95%CI=0.04, 0.34; p=0.06). |
| 11. | Results: Lines 364-371: It seems as if there were no significant interactions found between SDQ or YTP outcomes and baseline SDQ. Please make this clear, and also present the evidence supporting that there were differences between borderline/abnormal range SDQ at baseline and both SDQ and YTP outcomes (direct comparison, in support of your statement, with the acknowledgement that there is no significant interaction effect). | **Evidence supporting these differences was previously included in the manuscript draft in S4 and S5 Tables and described in the text on page 26-27. The relevant text has been slightly revised for clarity along with an explicit comment that the differences did not reach statistical significance, as below (page 26-27, line 406-413):**  For both primary outcomes, a stronger intervention effect was found for those scoring within the borderline range on the SDQ Total Difficulties scale at baseline, compared with cases scoring above the abnormal threshold at baseline (SDQ Total Difficulties, borderline range: AMD=-4.27, 95%CI= -7.62, -0.92; abnormal range: AMD=-0.53, 95%CI=-2.55, 1.50; p for interaction=0.06; YTP, borderline range: AMD=-1.79, 95%CI=-3.20, -0.38; abnormal range: AMD=-0.37, 95%CI=-1.21, 0.48; p for interaction=0.09). However, these differences did not reach statistical significance. |
| 12. | Results: Line 370: Please re-iterate the pre-specified variables here, and please clarify that you are still discussing moderation effects on the primary outcomes | **The pre-specified variables for moderation effects have been reiterated in Results section (page 27, line 413-415), as below:**  There was no evidence for moderation by any of the other pre-specified variables (baseline chronicity of mental health difficulties, YTP typology and SDQ caseness profile) on primary outcomes (see S4-S5 Tables). |
| 13. | CONSORT Checklist: Please complete the CONSORT checklist and ensure that all components of CONSORT are present in the manuscript. Please add the following statement, or similar, to the Methods: "This study is reported as per the Consolidated Standards of Reporting Trials (CONSORT) guideline (S1 Checklist)."  When completing the checklist, please use section and paragraph numbers, rather than page numbers. | **The suggested CONSORT statement has been included on page 9, line 150-151. The checklist has been uploaded with corresponding information about section/paragraph numbers (S2 Appendix).** |
| 14. | Table 1 and Table 2: Please define all abbreviations used in the table within the legend. | **Abbreviations have been included in the legends for both Tables, as below (page 22 and 25):**  **Table 1**  SDQ=Strengths and Difficulties Questionnaire.  YTP=Youth Top Problems.  PSS-4=Perceived Stress Scale 4-item version.  SWEMWBS=Short Warwick-Edinburgh Mental Well-Being Scale.  **Table 2**  SDQ=Strengths and Difficulties Questionnaire.  YTP=Youth Top Problems.  PSS-4=Perceived Stress Scale 4-item version.  SWEMWBS=Short Warwick-Edinburgh Mental Well-Being Scale |
| 15. | S4 Table and S5 Table: Please provide the p-values for all the intervention effects (only provided for Baseline Severity- Borderline row of Table S4). | **The objective of this analysis was to assess effect modification and the p-values shown in the final column are the relevant ones for the hypothesis being tested (i.e., no effect modification). Adding p-values for each stratum-specific effect will place undue emphasis on these effects, which does not align with the objective of our analysis. We have therefore opted not to include not a separate p-value for each stratum. If preferred by editors, we can remove the p-value for baseline severity of mental health difficulties from S4 and S5 Tables to make it consistent with other rows.** |
| 16. | S8 Table: We would suggest using adherence or “adherers” or similar rather than using compliance/complier | **To be consistent with our previous trial report, we have replaced “compliers” and “non-compliers” with “completers” and “non-completers” in S8 Table and its legend.** |
| **Reviewer 1’s Comments** | |  |
| 17. | Title: The comparator condition isn't mentioned in the title (as per PICOS), and I think it would be helpful to consider indicating it in some form, as the nature of the experimental comparison only becomes clear in the abstract. | **We have not made this change out of concern for making the title more cumbersome and difficult to follow. However, we will be guided by the editors regarding a suitable final title.** |
| 18. | p3, line 47: It may be helpful to state 'years old' after 15.61, so that it is clear what this statistic refers to. | **The word “years” has been added in the Abstract (Page 3, line 48), as below:**  Participants in the original trial were 251 school-going adolescents (Mean age=15.61 years, SD=1.68). |
| 19. | I was surprised to read that there were two primary outcomes - my understanding of having a primary outcome is that it is, by definition, singular, such that the problems of multiple testing are reduced. This may be a common procedure but I was, as someone relatively familiar with trial design, unfamiliar with it | **As described in the primary publication from the trial (Michelson et al., 2020), the rationale for having two primary outcomes was that problem solving would be directly relevant to managing an individual’s prioritized problems, with concomitant effects on a global measure of mental health symptoms. Previous studies have also included multiple primary outcomes in RCTs, so this is not an uncommon procedure if there is clear rationale. For details please see:**  **Vickerstaff V, Ambler G, King M, Nazareth I, Omar RZ. Are multiple primary outcomes analysed appropriately in randomised controlled trials? A review. Contemporary Clinical Trials. 2015 Nov 1;45:8–12.**  **We agree that it would be helpful to reiterate the rationale for including two primary outcomes, and so we have added the following detail to the Outcome measures section (page 13, line 244-246):**  As explained in Michelson et al. [7], two primary outcomes were included in the trial as we considered that problem solving would lead to reductions in prioritized problems with concomitant effects on mental health symptoms. |
| 20. | It may be useful to state in the abstract that this was an intention to treat analysis. | **We have updated the text in the abstract (Page 4, line 62), as below:**  …. using a linear mixed model for repeated measures, on an intention-to-treat basis. |
| 21. | From the abstract onwards (e.g., also on page 15), I was a bit confused by the difference between an analysis of outcomes AT 12 months and outcomes OVER 12 months. I assumed the latter was the difference from the baseline to the 12-month point, whereas the former was the raw comparison at 12 months, but this may be incorrect. Hence, it might be helpful to make this more explicit at some point in the paper, or use a more direct/explicit language (e.g., 'Change from baseline to 12-month assessment'). | **“At 12 months” refers to outcome analysis at the 12-month endpoint, while “over 12 months” refers to repeated-measure analysis assuming a constant effect at three follow-up points (6 weeks, 12 weeks and 12 months). We have clarified this distinction in both the abstract (page 3-4, line 60-62) and statistical analysis section (page 15, line 286-289), as below:**  **Abstract:**  The sustained effects of the intervention were estimated at the 12-month endpoint and over 12 months (the latter assumed a constant effect across three follow-up points) using a linear mixed model for repeated measures, on an intention-to-treat basis.  **Statistical analysis:**  In addition, the intervention effects on the SDQ, YTP and other secondary measures were analyzed over the 12-month period using repeated-measure analysis. This analysis used data from all three follow-up time points (i.e., at 6 weeks, 12 weeks and 12 months), assuming a constant effect across the time points. |
| 22. | p12, line 72. It didn't seem correct to claim that the evidence was for a sustained effect 'during' the 12-month period, as very little was known from the 12-week test to the 12 month endpoint test. 'Over' would seem a more accurate description of the sustained effect. | **In the Abstract (page 4, line 76), we have clarified that the evidence for sustained intervention effects was derived from analysis over 12 months, as below:**  … intervention had sustained effects on psychosocial problems and mental health symptoms over the 12-month follow-up period. |
| 23. | The per-student cost is made more salient in the paper, and is the cost provided in the abstract, whereas the per-case cost seems to me a more common and meaningful indicator of the actual economic cost of the intervention. To say that it costs $4 per school pupil to provide this intervention, when most of these pupils are not, in fact, receiving the intervention, does not feel particularly meaningful or precise to me. | **As described on page 9, line 156-159, a variety of whole-school and classroom “sensitization” activities were implemented to generate awareness of the available interventions in collaborating schools. Following this, referred students meeting the eligibility threshold were enrolled in the trial and randomized to one of two problem-solving interventions. Since the program costing provided in the manuscript covers all these components (see page 13-14, line 260-266), we feel that the per student cost is a more suitable indicator than per case for the actual and scale-up scenario.** |
| 24. | p.5, line 89. I was not familiar with term 'retrospective ingredients' approach. If this section is intended to be accessible to a non-specialist readership it may be advisable to use a more accessible or clearly descriptive term. | **Description of the retrospective ingredients approach has been added in the Costing Framework section (page 13, line 260-262), as below:**  A retrospective ingredients approach [15] was used to develop an intervention costing framework based on information obtained from the provider perspective after completing the trial. This included retrospectively mapping various activities, resources and time allocation used in setting up and implementing the intervention during the trial, with costings derived from records provided by the implementing organization, Sangath. |
| 25. | p9, line 166. It wasn't clear whose assent is being referred to here: adolescents or parents/carers. | **The assent process has been clarified, as below (page 10, line 179-186):**  One week prior to the 12-month follow-up date, attempts were made to establish telephone contact with the original trial participants in order to gauge their interest and availability for participation in the long-term follow-up study. Contact was also made with parents/guardians (‘caregivers’) for adolescents aged less than 18 years. If agreed, a face-to-face appointment was scheduled at the individual's home or in school, as per their preference. Written assent (or consent for those aged 18 years or older) was obtained from adolescents, followed by consent from a caregiver for adolescents aged below 18 years. |
| 26. | p12, line 198. Where was the reason for noncompletion from? Was this self-report or otherwise? | **Reasons for non-completion of the intervention were derived from a checklist completed by counsellors; this detail has been added to Page 11-12, line 217-218. We also note that the descriptive results on non-completion mistakenly specified 28 non-completers rather than 27. We have made the necessary corrections in the same paragraph (page 11-12, line 216-220) and in Fig 1:**  Ninety-eight (78%) participants completed the intervention (i.e., attended four or more sessions) [7]. Reasons for non-completion for the 27 participants was documented by counsellors using checklist with pre-specified categories and included: rapid resolution of the identified problems (n=13, 48%); competing demands at school (n=6, 22%); persistent absence from school (n=5, 19%); and unspecified (n=4, 15%) (Fig 1). |
| 27. | p13, Although details of the primary outcome measures are given in the original paper, I think it would be helpful to describe just a bit more here, particularly as the format of the YTP is somewhat non-standard/idiographic | **We have elaborated on the description of the primary outcome measures (page 12-13, line 236-243), as below:**  Two primary outcomes were assessed using self-report measures at 12 months after randomization: (i) mental health symptoms measured using the SDQ Total Difficulties score (range: 0 to 40) [9], which is derived by summing 20 items covering both internalizing and externalizing symptoms; and (ii) psychosocial problems, measured using the Youth Top Problems score (YTP, range: 0 to 10) [11], which is an idiographic measure for which an overall score is derived by averaging individual ratings for up to three prioritized problems nominated by the respondent. Higher scores indicated greater severity of symptoms and psychosocial problems respectively. |
| 28. | p14, Can a little more be given about the nature of the self-report measure of PS materials and skills: how were the questions phrased here? | **We have updated the text in the Outcome measure section (Page 13, line 255-258) to include English translations of the two self-reported questions that assessed use of problem-solving materials and skills respectively, as below:**  Additionally, we used a four-point Likert scale to collect data on adolescents’ self-reported use of problem-solving materials (“In the past year, how often have you used the booklets that you received during the program?”) and problem-solving skills (“In the past year, how often have used the skills that you learned during the program?”). |
| 29. | p20, I wonder if there is a need for the detail on the primary caregivers' education and occupation, or whether this might go into an appendices to shorten the paper somewhat. | **We have retained the same format as the primary trial paper to make categories comparable. We are willing to make this edit and will be guided by the editors’ final decision.** |
| 30. | p23, line 339-343: again, I struggled somewhat to understand what the statistic being given here was, as compared with the comparison of outcomes at 12 months. Is this the change from baseline? What does it mean 'SDQ = -1.23'? (I wonder if this is a typo and should read: 'SDQ: AMD =' | **As mentioned in response to comment 21 above, we have clarified in the text that “At 12 months” refers to outcome analysis at the 12-month endpoint, while “over 12 months” refers to repeated-measure analysis assuming a constant effect at three follow-up points (6 weeks, 12 weeks and 12 months).**  **We have also slightly amended the wording of the SDQ and YTP analysis to clarify the meaning of the statistics and incorporated the missing statistic term for SDQ, as below (page 23, line 370-376):**  The average difference in YTP score between the two arms at the 12-month endpoint was 0.75 points, in the direction favoring the intervention arm (AMD=-0.75, 95%CI=-1.47, -0.03; d=0.27, 95%CI=0.12, 0.42; p=0.04). Assuming a constant intervention effect across three follow-up time points, there was strong evidence of an intervention effect on both SDQ Total Difficulties and YTP scores over 12 months (SDQ: AMD=-1.23, 95%CI=-2.37, -0.09; d=0.21, 95%CI=0.05, 0.36; p=0.03; YTP: AMD=-0.98; 95%CI=-1.51, -0.45; d=0.34, 95%CI=0.19, 0.50; p<0.001) (Table 2). |
| 31. | I think more could have been said of the fact that neither the use of the booklet, or of problem solving skills, significantly mediated the outcomes. It states on page 31, line 427, that the findings strengthen the evidence base for the effects of problem solving, but without any evidence of a mediating effect, and given that the comparison condition was also problem solving (without counsellor input), it might be argued that the findings do not particular support the value of problem solving practices, per se but rather contact with a counsellor (for instance, as a consequence of relational common factors). Having said that, it is mentioned that the power was not sufficient for this mediation analysis and, if it is indeed the case that nothing can be gleaned from this null finding due to under-powering, I think it would be important to state that earlier on. | **We agree that it is not possible to discern the specific mechanism of the intervention effect in the absence of a fully-powered mediation analysis. We have incorporated this point into the discussion and also referred to a forthcoming process evaluation (page 30-31, line 469-479), as below:**  These findings strengthen the evidence base for the longer-term impacts of brief transdiagnostic interventions [20]. The study also adds to the growing evidence that adequately trained and supervised non-specialist providers may play a specific role in optimizing the effectiveness of psychological interventions for common mental health problems, and this strategy can help to overcome supply side barriers to scaling up evidence-based mental health care in low-resource settings [21,22]. However, it is not possible to discern the specific mechanism of the intervention effect in the absence of a fully-powered mediation analysis. Qualitative exit interviews with trial participants will be the focus of a future trial report and may shed more light on the potential role of problem solving as an active ingredient in driving therapeutic change. |
| 32. | S8 Table. It might be the formatting, but I struggled to understand what the means were that were being presented here. Is the data definitely in the right boxes? | **Table S8 has been reformatted so that the cell values align with row and column labels.** |
| **Reviewer 2’s Comments** | |  |
| 33. | Line 226-227: Are SDQ scores of 19 and 20 considered the clinical threshold here, as they were considered the cut-off scores for inclusion? | **We have clarified that the clinical thresholds for remission correspond to the trial’s original inclusion criteria for clinical severity, as below (page 13, line number 253-254):**  …. proportion of remitted cases (assessed using the ‘crossing clinical threshold method’ [14] and defined as scoring below clinical eligibility thresholds on the SDQ Total Difficulties and Impact scales [8]) |
| 34. | Lines 254-255 are not clear. The sentence describes adjustment for baseline measure, school, and missing FU, but the parenthetic (which seems to specify the particular variables adjusted for) includes age, class, and week of enrollment and do not include not missingness. | **In Statistical Analysis, we have clarified the sentence about baseline adjustments, as below (page 14, line 284-286):**  Analyses were adjusted for the baseline value of each outcome measure, school and variables associated with missingness at any time point (class, age, week of enrolment, baseline YTP mean score). |
| 35. | Discussion  How much variability is expected in cost of the intervention through other implementers? It would seem that other NGOs or governmental bodies would be needed to provide scale-up, at least beyond the 20 schools modeled. It would be helpful in considering the larger-scale impact if the authors could comment on how costs may vary in the paragraph starting at line 451. | **We have updated the Discussion section (page 32, line 504-512) to consider issues around variability in costs, as below:**  By hiring full-time school counsellors and supporting the costs of their training and supervision, school authorities could provide coverage for adolescents who might not otherwise receive services due to barriers such as transportation, cost or perceived stigma [25]. In doing so, it is important that the school authorities carefully plan the allocation of counsellors’ time, as the personnel cost was the biggest cost driver in the per-student cost estimate. Whereas a relatively large school might require more than one counsellor, several smaller schools could conceivably share one full-time counsellor to ensure more efficient use of resources. Given these cost variabilities, the per-student costs reported in this study should be considered indicative. |
| **Reviewer 3’s** **Comments** | |  |
| 36. | 1. Sample size. There is no sample size calculation in the paper which is inadequate. The original trial was designed for 6 and 12 weeks outcomes but modified to 12 months, why? and on what bases? However, as written in the paper "the sample size calculation did not take into account the numbers expected for the 12-month assessments", it means the current trial was not properly powered so would not be able to answer the research questions with confidence. | **As described in the Introduction section (page 8, line 128-138), the trial was originally designed to evaluate outcomes at 6 and 12 weeks. The relatively short follow-up periods were consistent with the conceptualization of the intervention as the first step of a stepped care architecture. However, findings from the 12-week follow-up indicated that the difference in symptom trajectories between the intervention and control arms was widening (see Michelson D, Malik K, Parikh R, Weiss HA, Doyle AM, Bhat B, et al. Effectiveness of a brief lay counsellor-delivered, problem-solving intervention for adolescent mental health problems in urban, low-income schools in India: a randomised controlled trial. The Lancet Child & Adolescent Health [Internet]. 2020 Jun 22 [cited 2020 Jul 13];0(0). Available from:** [**https://www.thelancet.com/journals/lanchi/article/PIIS2352-4642(20)30173-5/abstract**](https://www.thelancet.com/journals/lanchi/article/PIIS2352-4642(20)30173-5/abstract)**).**  **It was speculated that a longer follow-up period might have detected a distal effect on mental health, perhaps as newly learned coping skills were practiced and consolidated over time. A protocol modification was made to examine trajectories of trial outcomes over a longer 12-month assessment period. As this was a post hoc follow-up, no sample size calculation was taken for this follow-up. This was previously acknowledged as a limitation in the Discussion section in the manuscript. We have slightly modified wordings of this paragraph to make it clear that this was post-hoc addition (page 32, line 513-516), as below:**  We acknowledge a number of limitations to the study. First, the 12-month follow-up was a post-hoc addition to the originally scheduled follow-up assessments at six and 12 weeks. Consequently, the sample size calculation did not take into account the numbers expected for the 12-month assessments.  **We have also included additional information about the original sample size estimation in the Study design and participant section (page 9-10, line 169-173), as below:**  The estimated sample size for the originally stipulated primary endpoint assumed loss to follow-up of 15% at six weeks (based on piloting) and a 1:1 allocation ratio, to provide over 90% power to detect an effect size of 0·5 (and 80% power to detect an effect size of 0·4) at the α-level of 0·025 for each of the two primary outcomes [8]. Based on these estimates, a total of 251 participants were initially enrolled and 125 were randomly allocated to each arm after accounting for one participant in the intervention arm who withdrew consent after randomization. |
| 37. | Loss of follow-ups. About 30% of loss of follow-ups is of a big concern. Not matter how data was imputed and sensitivity analyses were done, the reliability and robustness of the results are subject to scrutiny. | **We previously acknowledged the loss to follow-up (LTFU) rate as a limitation in the discussion section and placed this in the context of other trials involving young people with common mental health problems (see page 32-33, line 516-519). We have reproduced the relevant text below for ease of reference:**  The observed LTFU rate at 12 months (29.4%) was higher than assumed for the original endpoints. That said, the 12-month LTFU was similar to the attrition rate at long-term follow-up in other randomized trials of interventions for youth anxiety and depression [26,27].  **Further, we have tried to address the possible bias with LTFU by adjusting for factors associated with missingness and with multiple imputation, which provided broadly consistent results, indicating the reliability of findings (S3 Table).** |
| 38. | Adherence to intervention. In the intervention arm, there are 97 completed & 28 discontinued intervention, therefore only 97 are per protocol with around 22% of those in the treatment arm didn't complete the intervention. However, this issue was never discussed in the paper or addressed in the analyses. | **We agree that intervention adherence is an important consideration when interpreting the results of the trial. Accordingly, the intervention non-completion rate and reasons for non-completion were presented on page 11-12, line 216-220 in the Intervention and control arms section. Differences in outcomes for completers and non-completer were also given in the Results section (page 27, line 419-423) and in S8 Table. We reflected further on these results in the Discussion section (page 31, line 480-490).** |
| 39. | Statistical analyses. The main results were shown in table 2 but very confusing. As shown in table 2, the control arm has 95 and intervention arm has 81 participants which shows this is complete case analysis rather than the 'intention to treat' analysis claimed by the authors. Also, the section on 'over 12 months' with 339 controls and 325 interventions is very confusing as it's never specified in the stats analysis section or anywhere. It should be outcomes at 12 months only in table 2. | **We have clarified the distinction between “at 12 months” and “over 12 months” as set out in our response to comment 21 above.**  **We would also like to clarify that this is a complete case analysis, but we have used intention-to-treat principles, e.g., analysing according to arm in which participants were randomized, and adjusting for factors associated with missing data. We did not impute for the primary analysis (but did do this for our sensitivity analyse, presented as supplementary materials noted above). The clarification for ‘n’ in Table 2 has been added to the table legend (page 25).** |
| 40. | Interpretation of results. What we can see from table 2 main analysis on the 12-month outcomes and also sensitivity analysis from the S3 table, there were either no differences or borderline differences in both primary or secondary outcomes between intervention and control arms. Coupled with inadequate sample size, 30% loss of follow-ups and 22% non-adherence to protocol (intervention arm), there is no evidence with sufficient power to support authors' claim on the usefulness of the intervention. | **As shown in Table 2 (page 24-25), a significant intervention effect (p<0.05) was found on most primary and secondary outcomes. We conducted sensitivity analyses (S3 Table) adjusting for missing data, which demonstrated results mostly consistent with our primary analyses and little evidence of differential loss-to-follow-up (LTFU). We have adjusted outcome analysis for factors associated with LTFU.** |
| 41. | Table 1 should be on comparing two arms rather than on loss of follow-ups, and it's also not properly done. For categorical variables, the percentage should be done column-wise rather that row-wise so that can make sense.  Not able to comment on the costing models as need specialist on this part. | **Post-hoc 12-month follow-ups were recommended by the Trial Steering Committee and agreed by the DSMB after unblinding the results of the originally stipulated 6- and 12-week follow-ups. The purpose was to examine whether the observed trend of a widening difference between the two intervention arms was sustained over time. Table 1 (page 19-22) examines to what extent those who took part in this follow-up study were similar or dissimilar from those who were lost. Accordingly, we have adjusted for factors associated with LTFU in further analysis. The rationale for row-wise percentages was to compare the LTFU proportion by exposure categories of each variable. But if editors feel column-wise percentages would enhance readability, then we would be pleased to make such changes.**  **The comparison of baseline demographic and clinical characteristics for two arms is presented in the primary trial paper. These were found to be similar across the two study arms (Michelson et al., 2020). It can be reproduced here if deemed necessary by the editors.** |
