# Supplementary material for: Effectiveness and costs associated with a lay counselor–delivered, brief problem-solving mental health intervention for adolescents in urban, low-income schools in India: 12-month outcomes of a randomized controlled trial
Source: PLoS Med. 2021 Sep 28;18(9):e1003778. doi: 10.1371/journal.pmed.1003778 (PMC8478208; doi:10.1371/journal.pmed.1003778)
Supplement: Attachment — Submitted filename: Editorcomment_July.docx [file pmed.1003778.s012.docx]

**Authors’ Responses to editors’ and reviewers’ comments**

|  | **Editors/ Reviewers Comments** | **Authors’ responses** (The page and line numbers correspond to the tracked version of the revised manuscript draft) |
| --- | --- | --- |
| **Editors’ Comments** | | |
| 1. | How did the authors assess fidelity of lay counsellors to the intervention? This is important if lay counsellors are used and scale-up is recommended in a context where mental health resources for adolescents are limited and lay counsellors are needed for program delivery. | **We have added a brief description of the procedures used to assess the quality of intervention delivery. Additional details can be found in the primary publication from the trial (Michelson et al., 2020). The revised text (Page 14, lines 232-236), is as below:**  **Weekly supervision was provided in a peer group format, which was moderated by a rotating counsellor and overseen by a masters-level or doctoral-level psychologist. Audio-taped sessions (one or two per week) were reviewed and rated on a number of key competencies with corrective feedback provided as appropriate; further details are described elsewhere [7].** |
| 2. | Please address the remaining points of the reviewers. Given Reviewer 3’s remaining concerns with the limitations of the study, please acknowledge these limitations as thoroughly as possible, and temper or qualify claims (in the Abstract and Discussion) in light of these limitations, where appropriate. | We have addressed Reviewer 3’s remaining concerns in our detailed responses to comments numbered 28-30. |
| 3. | Response to reviewer comments point # 41: It would be helpful to have the table of baseline demographic and clinical characteristics for the two arms. Thank you for noting that this information for the two arms was presented in the primary trial paper (Michelson et al, 2020). Although it can be found elsewhere, it would be helpful to include here as well for ease of access. | Thank you for this suggestion. We have added a table summarizing baseline demographic and clinical characteristics for the trail participants (labelled as Table 1, **pages 12-13**). The previous table showing characteristics of participants who were lost to follow-up has now been moved to the Appendix (S2 Table). |
| 4. | Data availability statement: Thank you for providing link for accessing the de-identified data. If possible, please provide additional information, or a more direct link, for accessing the specific data included in this study. | The link for the webpage is as follows:  [**https://doi.org/10.17037/DATA.00002147**](https://doi.org/10.17037/DATA.00002147) |
| 5. | Abstract Line 55-56: If helpful, you could clarify here where you describe that “A protocol modification was made…” that “the trial steering committee recommended a post-hoc extension of the follow-up period to 12 months” or similar language (to provide some context for the 12 month outcomes). | The suggested text insertion has been made to the abstract (page 3, lines 54-56), as below**:**  **The protocol was modified, as per the recommendation of the Trial Steering Committee, to include a post-hoc extension of the follow-up period to 12 months.** |
| 6. | Abstract: Methods and Findings: Please include a sentence summarizing adverse events, if relevant. | We have added the following sentence summarizing adverse events (page 4, lines 66-67):  **One adverse event was identified during follow-up and deemed unrelated to the intervention.** |
| 7. | In the last sentence of the Abstract Methods and Findings section, please describe the main limitation(s) of the study's methodology | **We have added the following sentence on the main study limitation** (pages 4-5, lines 82-83):  **The findings need to be interpreted with caution, as this study was a post-hoc extension.** |
| 8. | Author summary: Why was this study done? For this section, it may be helpful to include an introductory point on the broader context for the study (beyond mentioning the previous report on the trial findings). | We have added a bullet point providing more context to the study (page 6, lines 88-93), as below:  **• “PRIDE” (PRemIum for aDolEscents) is a transdiagnostic, stepped care program designed to address common adolescent mental health problems (anxiety, depression and conduct difficulties) in low-resource settings. It comprises a brief problem-solving intervention (“Step 1”), followed by a higher-intensity personalized psychological treatment (“Step 2”) for adolescents with persistent problems.** |
| 9. | Results: Line 401-402: Please present the results in the text for the intervention effect on SDQ externalization symptom scores over 12 months, although you note this did not reach statistical significance. | The results for the SDQ Externalizing symptoms subscale and remission have been reiterated in the text of the Results section (page 26, lines 419-422), as below:  **There was no evidence for an intervention effect on the SDQ Externalizing symptoms subscale score (AMD=-0.47, 95% CI=-1.09, 0.14; d=0.14, 95%CI=0.01, 0.30; p=0.13) and on remission over 12 months (AMD=1.47, 95% CI=0.73, 2.96; p=0.28).** |
| 10. | Results: Line 404: We suggest revising to clarify that while some of the effects for SDQ total difficulties no longer reached statistical significance in the sensitivity analyses, the effect did not appear to be attenuated for the sensitivity analysis adjusted for missing data over 12 months. | **The text has been clarified as suggested on** page 26, lines 422-426, and reproduced below:  **The intervention effect on most outcomes was relatively stable across sensitivity analyses, except for the SDQ Total Difficulties and SDQ Impact scores. The effect on these two outcomes no longer reached statistical significance in the sensitivity analysis adjusting for missing data, and the effect was slightly attenuated compared with the primary analysis (S3 Table).**  **We have additionally referred to findings of a weaker SDQ effect in sensitivity analysis in the Abstract and Discussion sections, as below:**  **Abstract (page 4, lines 73-74)**  **The intervention effect was stable for most outcomes on sensitivity analyses; however, for SDQ Total Difficulties and impairment, the effect was slightly attenuated**  **Discussion (page 30, lines 484-488), as below:**  **Compared with previously reported post-intervention outcomes at six and 12 weeks [7], our findings indicated sustained intervention effects on both psychosocial problems and mental health symptoms over 12 months; however, evidence for impact on mental health was weaker in the sensitivity analysis that adjusted for missing data.** |
| 11. | References: Please ensure the use of the "Vancouver" style for reference formatting (including Journal title abbreviations), and see our website for other reference guidelines <https://journals.plos.org/plosmedicine/s/submission-guidelines#loc-references> | All the references have been checked and updated to comply with the Vancouver style of formatting. |
| 12. | Figure 2a and 2b: Please mention in the legend that the error bars represent mean with SD, if accurate. Please also fully define abbreviations YTP and SDQ in the legend. | As per the PLOS submission guidelines, we previously included the figure 2’s legend and the caption in the main text file (below first paragraph in the effectiveness subsetction). As per editors’s suggestion, we have also now included the same text insertion to the legend of Figures 2a and 2b. |
| 13. | Table 2: Please note if values are reported as mean score with (SD), if accurate. | We have updated column headings in Table 2 (Page 23) to indicate where mean scores (with SD) apply. |
| 14. | CONSORT checklist: Thank you for including the CONSORT checklist. Please remove references to page numbers, and instead use sections and paragraph numbers to refer to locations within the text. For “Funding” we suggest reporting the location as “Financial Disclosure statement” or similar. | The CONSORT checklist has been revised to include the section and paragraph number for each item. The funding item on the list has also been revised as per the editors' suggestion |
| **Reviewer 1’s Comments** | | |
| 15. | Point 21: I continue to feel that the use of the term 'over' 12 months, as opposed to 'at' 12 months, is not as clearly defined as it could be across the MS. It may be that this is common terminology that I am not familiar with and, if so, I am happy to leave this point; but as an informed reader I was still left a bit uncertain what this meant particularly, for instance, on the data presented in Table 2. I think I now understand that the 'over 12 months' score is the predicted score based on the three timepoint, assuming linear change (as opposed to the exact score at 12 months). In the abstract this is explained as, 'the latter assumed a constant effect across three follow-up points'. That gets me closer to what is being described here, but it still doesn't quite tell me WHAT the score is (for instance, is it the predicted 12 months score based on the assumption of a constant effect, or the change over time assuming a constant effect?). From Table 2 it seemed to be the former, but 'over' just doesn't feel like the right word here. 'Predicted' or 'estimated' might be clearer? | We wish to clarify that we have modelled the outcomes in two ways: for our primary outcome analyses, these are modelled ‘at 12 months’, which refers to the scores at that time-point; for the secondary outcome analyses, we have modelled all the ratings for each participant across the 12 months (i.e., at three follow-up time points of 6 weeks, 12 weeks and 12 months) using repeated measures analyses.  We have added text in the Statistical Analysis section to clarify the meaning of the “over 12 months” analysis (page 18, lines 311-317), as below:  **In addition, for secondary outcome analyses, the intervention effects on the SDQ, YTP and other self-reported measures were analyzed over 12 months using similar repeated-measures analysis. This analysis used data from all three follow-up time points (i.e., at 6 weeks, 12 weeks and 12 months), assuming a constant effect across the time points. Thus, analysis “over 12 months” represents the estimated effect size at each time point and more generally throughout the 12 months follow-up period.** |
| 16. | Point 23: Although I can see the authors' point here, there seems something of an incongruity to me to be presenting all clinical change (in the abstract) on a per-case basis, but then the costs on a per-student basis. If the principal concern were to look at the whole school effects, surely the mental wellbeing outcomes should be per student as well. Perhaps a 'compromise' here would be to provide the per participant costs along with the per-student costs in the abstract: for instance in parenthesis after the USD4. | **The per-case cost has been added to both Abstract and Discussion sections, as below.**  **Abstract (page 4, lines 75-76):**  **The per-student cost of scaling up the intervention was USD 4 (or USD 23 per-case), which accounted for 0.4% of the per-student school budget in New Delhi.**  **Discussion (page 32, lines 538-541):**  **Our economic evaluation indicated that a brief, lay counsellor-delivered problem-solving intervention could be scaled up at the cost of USD 4 per student (or USD 23 per-case), which is a small fraction (0.4%) of the per-student budgetary allocation in government run-schools in New Delhi, India [19].** |
| 17. | Line 209: It's just a little confusing that it talks about the people who delivered the intervention, and then subsequently the counsellors. I'm 99% certain, as a reader, that this is the same group, but perhaps just change 'The counsellors' to 'These counsellors' on line 201-211 to make clear it's the same people. | The relevant text on page 14, lines 230-232 has been clarified as below:  **These counsellors were employed by the implementing organization (Sangath) and trained through a combination of office-based learning and supervised field practice.** |
| 18. | Line 245: It's good to have some explanation of why there are two primary outcomes. However, it does then beg the question of whether you looked to see any cross-lagged effects from changes in prioritized problems to effects on mental health symptoms. I couldn't see any of that analysis here. Is it something to suggest for future research? I guess that depends on how strongly you hold this hypothesis. If just a loose guess, it might make more sense on lines 244-246 to talk about these as two important outcomes rather than suggesting a causal relationship. | **As suggested by the Reviewer, we have revised the description of the two primary outcomes so that there is no suggestion of causality/cross-lagged effects**. The revised text (page 16, lines 268-271) is reproduced below:  **As indicated in Michelson et al. [7], two primary outcomes were included in the trial as we considered that problem solving would lead to reductions in both prioritized problems (an adolescent-centered outcome) and mental health symptoms (a standardized outcome).** |
| 19. | Table 2: Apologies if I missed this, but I wasn't sure why you had the 'at' and 'over' AMDs/ESs/ps for the two primaries, but then not for the secondaries. Do these need adding in too? If not, are the data given 'at' or 'over'? | As mentioned in the Statistical Analysis section (page 18, lines 311-313), the intervention effect on all secondary measures (except remission) was analyzed over 12 months. To clarify the secondary analysis timepoint, we have added the term “over 12 months” in the rows for secondary outcomes in Table 2 (page 23-25). |
| 20. | Line 429 says that the per-case cost was USD 158, but this seems to reduce to USD18 at line 446. Is a 'per-student treated' cost the same as a 'per-case' cost (if so, please align terminology and, if not, please explain the former term). More importantly, I did struggle to understand how the cost when the intervention was scaled up was reduced by almost 90%. Are those calculations definitely right? (I checked S3 but that didn't really help explain the magnitude of reduction). Perhaps a few added words in the text here might help the reader to understand why the cost becomes so much less. | **Concerning the Reviewer’s query regarding per-student cost and per-case cost: the former refers to the average cost of the program per student enrolled in the school, and the latter refers to the average cost of the program for each case treated in the program. These have been elaborated as footnotes to Table 3 (page 29, lines 477-480), as below:**  **^9^ Indicates per capita cost; calculated by dividing the per-school cost by the average number of students in each school**  **^10^ Indicates cost for each treated case calculated by dividing the per-school cost by the average number of cases treated in each school**  **Concerning the Reviewer’s query about reduced scale-up costs: as mentioned in the Cost Analysis section of the manuscript draft, we modeled the hypothetical scale-up costs by considering the number of working days, types of program activities, training and supervision based on data available from the implementation of the original trial. We further elaborated on these assumptions in Appendix S3. There we have indicated that in the scale-up scenario, each counsellor will engage exclusively in activities that are directly related to the problem-solving intervention. Hence, each counsellor in the scale-up scenario will provide the intervention to a larger number of students in an academic year than was the case for trial counsellors. In the trial, the research procedures and restrictions on the number of adolescents recruited from each school for trial activities made it difficult for counsellors to optimally utilize their time in schools, leading to increased per-case cost.**  **With the increase in the potential caseload, the proportion of materials cost associated with each school in scale-up was higher, as compared to the trial, and thus, the slightly increased per-school cost. However, with this capacity to take an increased number of cases, the proportion of personnel cost per-case was reduced, and therefore, lowering the per-case cost as compared to the trial.**  **We have updated the relevant text from Appendix S3 and incorporated this into the main manuscript so that the above-mentioned assumptions are set out more clearly for the reader.** The revised text (page 20, lines 354-362) is reproduced below:  **In modelling the requirements of the hypothetical scale-up, we used findings from the original trial report [7], as related to the number of working days, types of program activities (sensitization, screening and intervention), the time required for each activity, and time needed for training and supervision. Additionally, we assumed that each counsellor would work full-time and engage exclusively in activities related to the problem-solving intervention over the duration of one academic year (not counting holidays). We also assumed that each counsellor’s available time in school would be fully occupied by referred cases (i.e., there would be no shortfall in demand).**  **In the Results section, we have revised the text to include the possible caseload for the scale-up scenario and the associated per-treated case cost.** The revised text (page 28, lines 464-470) is reproduced below:  **We estimate that in each school, a counsellor would be able to provide the intervention for up to 189 eligible students, affording a larger caseload than was possible for each counsellor in the trial. This corresponds to 3,780 cases across 20 schools in an academic year with a per-case cost of USD 23 (S3 Appendix). The higher caseload in the scale-up model led to a higher material costs, accounting for 25% of the total cost, compared to 3% of the total cost during the trial.** |
| 21. | Table 3: The per-treated case cost is now 23 USD. Should that be 18, as above. I really failed to understand the table here. Is there some way it can be made clearer? How is it that the Total cost in the top row is not the total of the subsequent costs? Why is the per-school cost greater in the scale up version than for the six schools, but the pre-treated case cost so much lower? As a reader (albeit not expert in economic analysis), this table, and the summary of it (lines 434-446) could do with some further work to really bring out the key findings and the logic of the analysis. | **The total cost in Table 3 (page 29) is the sum of the set up and implementation costs. The next three rows in the table show the bifurcation of total cost into staff, materials and capital costs. The last three rows represent the school-wise costs. We have added headings and footnotes** to clarify the respective meanings of the various cost categories. The footnotes (line 473-476) are reproduced below:  **^7^Component wise breakdown of total cost into staff, material (e.g., printed sheets) and capital costs (e.g., furniture)**  **^8^ Indicates cost for each school to set up and implement the program in an academic year; calculated by dividing the total cost by the number of schools enrolled in the program**  **Concerning the Reviewer’s query about why the** per-school cost is greater in the scale up scenario, we have provided the explanation for the same in our response to the preceding comment (#20). |
| 22. | lines 559-562: It seemed strange to end the paper by referencing second line interventions. No evidence was presented to support the claim that this would improve effects, and it seemed to take away from the value of the first-line intervention effects being reported here | We have revised the last sentence to indicate that future research could usefully examine the incremental benefits of supplementing the brief first-line problem-solving intervention with a more intensive treatment. This fits with the original rationale for the first-line intervention, which we have reiterated in the author summary (see response to comment 8 above). The revised sentence (page 35, lines 596-599) is reproduced below:  **Future evaluations are needed to examine whether incremental benefits can be achieved by supplementing a first-line problem-solving intervention with a more intensive treatment as part of a stepped care model.** |
| **Reviewer 2’s Comments** | | |
| 23. | I am either confused by the presentation or there is an error in the data entered in Table S8. How did completers have a baseline SDQ mean of 8? Wouldn't it have had to be higher than 19, considering that was the cutoff for inclusion? Is baseline at the end of the treatment and not the beginning of the trial?  It looks like something may be wrong with the YTP cells as well, with completers having a mean score of 1.5 at baseline and 2.4 at follow-up while non-completers have baseline of 7.1 (much closer to the mean baseline YTP score in Table 1). Neither of these low baseline scores seem reasonable when looking at Figure 2 graph means and error bars. If these numbers are in fact correct, the authors need to clarify how the SDQ baseline mean was 8 (far below the inclusion cutoff) and why completers' baseline scores for both the SDQ and YTP were so low compared to non-completers. If they are incorrect, in addition to updating results text and the table, the authors need to change the discussion paragraph on dose-response to reflect the correct finding. | We thank the Reviewer for bringing issues related to S8 Table to our attention. S8 Table has been reformatted with appropriate row and column labels. The descriptions in the Results and Discussion sections are correct and require no further changes. |
| 24. | I think there is not enough attention paid to the fact that the average symptom reduction over time and the percent of remitters in the control and intervention are quite similar. Yes, there is a significantly higher reduction in the intervention, but the AMD for the SDQ total difficulties is <2. Considering 1) the SDQ total scores can range from 0-40, 2) 19/20 is the clinical cutoff, and 3) both the intervention arm and control arm seem to have an average symptom reduction of ~10 points over the 12 months (Table 2B), I think the authors need to comment on the clinical significance of such a small difference between arms | **Recent research has shown that assessing change across multiple domains is more meaningful in understanding impact than focusing narrowly on symptom-based outcomes (Krause et al., 2021). In the current study, we observed a small effect on the SDQ Total Difficulties scale (a measure of global psychopathology),** comparable to the long-term outcomes achieved by more intensive interventions delivered by specialists. Moreover, this effect **was accompanied by a significant effect on the YTP (an idiographic measure of problems' meaningful to a young person) across three follow-up points. We also observed improvements on adolescent-reported measures of functional impairment, and perceived stress over 12 months. Looking at these findings in the aggregate, it can be said, with reasonable confidence, that the brief counsellor-led problem-solving intervention (total duration: 2-2.5 hours per child) achieved a small but meaningful impact in a public health context.**  **We have revised the text in the Discussion section to emphasize the array of outcome improvements, and particularly the impact achieved on the adolescent-centered YTP. The revised text** (page 30-31, lines 499-508), **is as below:**  **It is noteworthy that the longer-term effect of this brief, transdiagnostic, problem-solving intervention on mental health symptoms, although small, is comparable to the long-term outcomes achieved by more intensive interventions delivered by specialists [3]. Further, the effect on mental health symptoms was accompanied by a comparable effect on the YTP (an idiographic measure of problems' meaningful to a young person), as well as significant improvements in domains of functional impairment and perceived stress. These long-lasting effects across multiple domains strengthen the evidence base for brief transdiagnostic interventions [20], and indicate the potential for small but meaningful and sustained impact in low-resourced public health contexts [21].**  **Citation**  Krause KR, Edbrooke-Childs J, Singleton R, Wolpert M. Are We Comparing Apples with Oranges? Assessing Improvement Across Symptoms, Functioning, and Goal Progress for Adolescent Anxiety and Depression. Child Psychiatry Hum Dev [Internet]. 2021 Apr 7 [cited 2021 Jun 10]; Available from: <https://doi.org/10.1007/s10578-021-01149-y> |
| 25. | Moreover, while I appreciate the authors edits to remove emphasis on problem-solving and clarify the potential optimization by non-specialist providers as well as their mention that the study was not powered to detect mediators (lines 467-479), I think this needs to be more hashed out. Are the problem-solving booklets alone a good intervention? | **The Reviewer raises an important question about the potential merits of the problem-solving booklets as an intervention in its own right. In our original trial report, we noted that** research in high-income countries (e.g., Sharpe et al., 2016) has found minimal effects of booklets on mental health outcomes in school settings – although it is possible that such formats may be more potent in settings where alternative support is unavailable and expectancies may differ.  We have acknowledged the importance of exploring these issues in future research on **page 31 (lines 514-517),** as below:  **Qualitative exit interviews with trial participants will be the focus of a future trial report and may shed more light on the potential role of problem-solving skills as an active ingredient in driving therapeutic change, as well as considering the respective contributions of booklets and counsellors in the development of problem-solving capacity.**  **Citation**  Sharpe H, Patalay P, Vostanis P, Belsky J, Humphrey N, Wolpert M. Use, acceptability and impact of booklets designed to support mental health self-management and help seeking in schools: results of a large randomised controlled trial in England. Eur Child Adolesc Psychiatry. 2017 Mar 1;26(3):315–24. |
| 26. | Considering the majority of costs came from personnel, the control condition may actually be a more cost-effective intervention. Is it possible that there was contamination from adolescents in the intervention group discussing their treatment with peers in the control group? | **In our formative work, we identified that sharing of booklets/other printed materials was a more likely source of contamination than discussion of the counselling process per se (Michelson et al. 2020a). Indeed, this is part of the reason why booklets were chosen as the comparator in the current trial (i.e., contamination would be neutralized since participants in both arms received the same materials).** |
| 27. | I think there also may be a typo in lines 416-418 "There was no evidence that perceived stress, use of booklets of problem-solving skills at the 12-week endpoint mediated the intervention effect on primary outcomes at 12 months (S6-S7 Tables)." | The text has been corrected on page 27 (lines 437-439) as below:  **There was no evidence that perceived stress, use of booklets or problem-solving skills at the 12-week endpoint mediated the intervention effect on primary outcomes at 12 months (S6-S7 Tables).** |
| 28. | In sensitivity analyses adjusting for missingness, it appears that the SDQ impact score was not significantly different in the intervention vs. control over 12 months (Table S3). The authors should add to this sentence in addition to the mention of the weakened effect seen for the SDQ total difficulties score in the results and discussion sections | **This mirrors comment 10. As noted above, the text has been modified on** page 26 (lines 422-426) and is reproduced below.  **The intervention effect on most outcomes was relatively stable across sensitivity analyses, except for the SDQ Total Difficulties and SDQ Impact scores. The effect on these two outcomes no longer reached statistical significance in the sensitivity analysis adjusting for missing data, and the effect was slightly attenuated compared with the primary analysis (S3 Table).** |
| **Reviewer 3’s** **Comments** | | |
| 29. | Thanks authors for their effort to improve the manuscript. However, I am not satisfied with the response and revision at all. The authors failed to address almost every single one of my comments/concerns. Just to reiterate, there were either no differences or borderline differences in both primary or secondary outcomes between intervention and control arms. Coupled with inadequate sample size (not powered at all), 30% loss of follow-ups and 22% non-adherence to protocol (intervention arm), there is no evidence with sufficient power to support authors' claim on the usefulness of the intervention.  The study is not powered for the 12-month primary outcome at all. It looks more like an observational study with follow-up | We have very carefully considered the Reviewer’s original comments and their further feedback.  Regarding the point about difference between arms and inadequate sample size: Table 2 (page 23-24) shows that there were significant intervention effects (p<0.05) on all primary and secondary outcomes except for the SDQ Externalizing subscale score (p=0.13) and SWEMWBS score (p=0.06). Hence, there is evidence of significant differences by arm for the primary and most secondary outcomes. Given this, we respectfully disagree that the study is not powered for the 12-month primary outcomes. That said, we have already clarified in response to the first round of comments that the 12-month follow-up was a post-hoc addition to the originally scheduled follow-up assessments at six and 12 weeks. Consequently, the sample size calculation did not take into account the numbers expected for the 12-month assessments. We have recognized this as study’s limitation in both the Abstract and Discussion (see response to comments 7 and 30).  Regarding loss to follow-up: as stated in our first round of responses, we conducted sensitivity analyses adjusting for missing data, which demonstrated results mostly consistent with our primary analyses and little evidence of differential loss-to-follow-up. We further adjusted outcome analysis for factors associated with LTFU.  Regarding usefulness of intervention: As noted in our response to comment 24, the effect of this brief, transdiagnostic, low-cost intervention on mental health symptoms, although small, is comparable to the long-term outcomes achieved by more intensive interventions delivered by specialists. Further, the changes on mental health symptom were accompanied by a significant reduction in the severity of personally meaningful problems nominated by young people themselves (as assessed on the idiographic YTP), which is a more person-centered outcome of interest. These findings strengthen the evidence base for the sustained and meaningful impact of brief transdiagnostic interventions, particularly in low-resourced public health contexts. |
| 30. | The analyses are not 'intention to treat' at all. Please stop using it and acknowledge as a limitation carefully | We used intention-to-treat (ITT) principles (e.g., analyzing according to arm in which participants were randomized, and adjusting for factors associated with missing data) to conduct the primairy and secondary outcome analysis. However, we appreciate the Reviewer’s concern in labelling this as an ITT analysis, and have replaced the term “ITT” with “complete-case analysis” throughout the paper (i.e. in the abstract, methods and discussion sections). Additionally, in these sections, we have clarified that complete-case analyses were adjusted for missingness and random effects and sensitivity analysis using multiple imputation was carried out to address the risk of bias due to attrition.  Abstract **(pages 3-4, lines 59-63):**  **The sustained effects of the intervention were estimated at the 12-month endpoint and over 12 months (the latter assumed a constant effect across three follow-up points) using a linear mixed model for repeated measures, and involving complete-case analysis. Sensitivity analyses examined the effect of missing data using multiple imputations.**  Effectiveness analysis **(page 18, lines 305-308 and line 320-324):**  **Primary outcome analyses at 12 months were conducted using complete-case analysis. Analyses were adjusted for the baseline value of each outcome measure, school and variables associated with missingness at any time point (class, age, week of enrolment, baseline YTP mean score).**  **….**  **Sensitivity analyses examined the effect of missing data using multiple imputations with a linear imputation model (truncated at zero) adjusting for factors associated with missingness for the imputation (age, class, parents’ gender, baseline SDQ Total Difficulties and YTP scores, arm allocation) with 50 imputed datasets**  **Discussion (Page 33, lines 558-562)**  **To reduce risk of bias due to attrition while using complete-case analysis, variables associated with LTFU were adjusted in these analyses. Further, sensitivity analysis with missing data showed intervention effects were similar to the primary analysis for most variables except SDQ Total Difficulties and Impact scores at 12 months, where the effects were weaker.** |
| 31. | For RCT, it's not acceptable without a baseline table 1. | A table indicating baseline demographic and clinical characteristics for the trial participants has been added (Table 1, **page 12-13).** |
