# Supplementary material for: Effectiveness and costs associated with a lay counselor–delivered, brief problem-solving mental health intervention for adolescents in urban, low-income schools in India: 12-month outcomes of a randomized controlled trial
Source: PLoS Med. 2021 Sep 28;18(9):e1003778. doi: 10.1371/journal.pmed.1003778 (PMC8478208; doi:10.1371/journal.pmed.1003778)
Supplement: Attachment — Submitted filename: Editorscomment_Aug2021_rev.docx [file pmed.1003778.s013.docx]

**Authors’ Responses to editors’ and reviewers’ comments**

|  | **Editors/ Reviewers Comments** | **Authors’ responses** (The page and line numbers correspond to the tracked version of the revised manuscript draft) |
| --- | --- | --- |
|  | Requests from editors | |
| 1. | Title: We suggest revising the title to: “Effectiveness and costs associated with a lay counsellor delivered problem-solving mental health intervention for adolescents in urban, low-income schools in India: 12 month outcomes of a randomized controlled trial.” or please revise similarly with the aim of making the title as concise as possible. | **The suggested text insertion has been made to the Title (page 1, lines 1-3), as below:**  Effectiveness and costs associated with a lay counsellor delivered, brief problem-solving mental health intervention for adolescents in urban, low-income schools in India: 12-month outcomes of a randomized controlled trial |
| 2. | Data availability statement: Thank you for providing a link to access the de-identified data. We request that you provide more specific information to facilitate access to the data. Please provide a more direct link to the data, and/or include the DOI or accession number for the dataset. | **The ‘PRIDE 12-month outcome dataset’ that underpins the analysis in this paper are hosted in LSHTM Data Compass at** [**https://doi.org/10.17037/DATA.00002147**](https://doi.org/10.17037/DATA.00002147)**. In accordance with ethical constraints established when obtaining participant consent, data can only be made available to interested parties on the condition that they sign an agreement stating that they will protect participant confidentiality. To request access, please submit a data request at** [**https://doi.org/10.17037/DATA.00002147**](https://doi.org/10.17037/DATA.00002147) |
| 3. | Response to reviewer 1: Please address the comment of the reviewer regarding the mediation analysis and lack of power | **We have addressed Reviewer 1’s concerns on the power of mediation analysis in our detailed responses to the comment number 16.** |
| 4. | Response to reviewer 3: Please balance the description of results and interpretation in response to Reviewer 3’s points regarding the loss to follow up at 12 months and the borderline significance of some findings (namely the SDQ Total Difficulties Scale comparison at 12 months, taken together with the results from the sensitivity analysis).  The editors ask that acknowledgement of this limitation be given more explicit emphasis throughout where implications of the results are considered, particularly in the Abstract and Discussion. | **We have addressed Reviewer 3’s concerns on borderline significance of outcomes and LTFU in our detailed responses to comments numbered 17-18.** |
| 5. | Abstract: Line 74: “The intervention effect was stable for most outcomes on sensitivity analyses; however, for SDQ Total Difficulties and impairment, the effect was slightly attenuated.” Please clarify the sensitivity analyses mentioned here (that this was the sensitivity analysis adjusting for missing data) rather than just saying the sensitivity analysis showed an attenuated effect | **The suggested text insertion has been made to the Abstract (page 4, lines 73-75), as below:**  The intervention effect was stable for most outcomes on sensitivity analyses adjusting for missing data; however, for SDQ Total Difficulties and impairment, the effect was slightly attenuated |
| 6. | Abstract: As the last sentence of the Abstract Methods and Findings section, please describe the main limitation(s) of the study's methodology. | **We have added the following sentence in the Abstract outlining the main limitations of the study's methodology (page 4, lines 78-80):**  The main limitations of the study's methodology were the lack of sample size calculations powered for 12-month follow-up and the absence of cost-effectiveness analyses using the primary outcomes. |
| 7. | Abstract: Line 82-83: In line with reviewer 3’s comments, we think it may be helpful to provide slightly more general detail on why caution is needed given the post-hoc extension of the trial. We suggest also mentioning the specific limitations as the last sentence of the Methods and Findings section of the abstract. | **We have expanded on the last sentence in the Abstract to outline the main limitation associated with post-hoc extension (page 5, lines 86-88), as below:**  The findings need to be interpreted with caution, as this study was a post-hoc extension, and thus, the sample size calculations did not take into account the relatively high attrition rate observed during the long-term follow-up.  **We have addressed concerns related to specific limitations of study methodology in the comment 6.** |
| 8. | Discussion: Line 487-488: Referring back to Reviewer 3’s point, it may be helpful here to qualify the conclusions slightly, noting that the evidence supports an effect 12 months, but the effects are small particularly for SDQ Total Difficulties. | **We have amended the wording in the Discussion to clarify that the effect sizes were small, particularly for SDQ Total Difficulties, and its significance attenuated further during sensitivity analysis (page 31, lines 492-497), as below:**  Compared with previously reported post-intervention outcomes at six and 12 weeks [7], our findings indicated small but sustained intervention effects on psychosocial problems and mental health symptoms over 12 months. However, the intervention effect on mental health symptoms was borderline, which attenuated further in the sensitivity analysis that adjusted for missing data. |
| 9. | Tables: For the notes in the legend, we suggest not numbering the notes across tables, but instead numbering the notes beginning with “1” for each table. | The suggested changes in tables’ legend have been made on pages 25 and 29, and 30 |
| 10. | Table 3: Please provide the definition for abbreviation (USD) in the legend. | **The abbreviation USD has been fully defined in the legend of Table 3 (page 29, line 479).** |
| 11. | References: Please use the "Vancouver" style for reference formatting, and see our website for other reference guidelines: https://journals.plos.org/plosmedicine/s/submission-guidelines#loc-references | **All the references have been checked and updated to comply with the Vancouver style of formatting.** |
| 12. | Figure 1 Title and S2 Appendix: In the title of Figure 1 and for the title of S2_Appendix listed on page 41, please capitalize CONSORT. | **The relevant text in the title of Figure 1 (page 11, line 210) and heading of Appendix S2 have been capitalized (page 43, line 737), as below:**  Fig 1: CONSORT diagram for trial participants  S2 Appendix: CONSORT checklist |
| 13. | S8 Table: In the file and title name for this table listed in the main text (page 41), please change “compliers” and “non-compliers” to completers. Thank you for making this change within the table file. | **The suggested text insertion has been made to the heading of S8 Table in the main file (page 43, lines 754-755), as below:**   - S8 Table: Dose-response effect on primary outcomes at 12-month for intervention completers and non-completers |
| 14. | S3 Appendix: Please provide a clean version of the file (there is some mark-up in the reference to S1a, S1 b Tables). Please format the reference in this document similar to those in the main text (with the in text citation within square brackets rather than superscript, and the appropriate reference formatting). | **S1a-b Tables and S3 Appendix have been cleaned and we have included the relevant references using Vancouver style.** |
|  | Comments from Reviewer 1: | |
| 15. | Abstract: The actual per-case (and per-student) costs need to be given in the abstract, as this is the principal finding from the study (USD158). If the estimated/scaled up per case/per-student costs are also given in the abstract (USD4/23), as present, it should be made clear that this is an 'estimate'. | **In the Abstract, we have added the per-student and per-case cost for the trial and clarified that scale-up cost pertains to modelled scenario (Page 4, lines 75-77), as below:**  The per-student cost of delivering intervention during the trial was USD 3 (or USD 158 per-case) and that of scaling up the intervention in the modelled scenario was USD 4 (or USD 23 per-case). The scaling up cost accounted for 0.4% of the per-student school budget in New Delhi. |
| 16. | line 512-514. It needs to be made clearer here that the mediation effects of specific mechanisms/processes were tested, and not found to significantly mediate. This seems to be the first time that the lack of power for this analysis is given, and it would be helpful if this was stated earlier in the methods. An indication of the specific power for this analysis may be helpful if possible. | **We have clarified in the Discussion section that mediation effects were tested for specific hypothesized variables (Page 31, lines 503-505), as below:**  The observed intervention effect at 12 months was not mediated by any of the hypothesized variables i.e., perceived stress, use of problem-solving booklets and problem-solving skills.  **We have added details in the Statistical Analysis section to indicate that both mediation and moderation analyses were exploratory, as the sample size calculations were not powered for these analyses (Page 19, lines 334-341), as below:**  An exploratory, pre-specified effect moderation analysis tested for heterogeneity of intervention effects by the following: baseline chronicity of mental health difficulties, baseline severity of mental health difficulties, YTP typology and SDQ caseness profile. Post-hoc, SDQ caseness profile was removed from moderation analysis, due to the small number of observations (less than 6) in certain cells. In addition, exploratory mediation analyses were conducted to examine whether a priori factor of perceived stress, and two process indicators (use of problem-solving booklets and use of problem-solving skills) mediated the effects of the intervention on the primary outcomes in the longer term. |
|  | Comments from Reviewer 3: | |
| 17. | Thanks authors for their effort to improve the manuscript. The authors have addressed some of my concerns such as clarifying the 'intention to treat' and also adding the baseline Table 1. However, I am still not convinced/satisfied with the response to point 29 on sample size, power and statistical significance.  he authors said "Regarding the point about difference between arms and inadequate sample size: Table 2 (page 23-24) shows that there were significant intervention effects (p<0.05) on all primary and secondary outcomes except for the SDQ Externalizing subscale score (p=0.13) and SWEMWBS score (p=0.06)". However, out of 9 comparisons in table 2, there are 7 p-values ranging from 0.02 to 0.06 therefore means borderline significance (very close to 0.05) in 78% of all the results. Also the sensitivity analyses with missing data imputation didn't help. Basically the results in table 2 showed very weak and inconclusive evidence (if any) on the effectiveness of the intervention, which has never been acknowledged and discussed in the paper. | **As indicated in Table 2 (Page 23-25), of the ten primary and secondary outcomes, six were statistically significant (p<0.05), one was borderline (p=0.05), and three were non-significant (p>0.05). Thus, the Reviewer's assertion that most of the outcomes were borderline significant does not represent our reading of Table 2. Of course, we accept that the intervention effect sizes on these outcomes were small, and some did not reach statistical significance. We have clarified about the same in the Discussion section (Page 31, 492-500), as below:**  Compared with previously reported post-intervention outcomes at six and 12 weeks [7], our findings indicated small but sustained intervention effects on psychosocial problems and mental health symptoms over 12 months. However, the intervention effect on mental health symptoms was borderline , which attenuated further in the sensitivity analysis that adjusted for missing data. There was also evidence for longer-term effects on internalizing symptoms, distress/functional impairment, perceived stress and well-being. Similar to the results obtained at six and 12 weeks, we found no evidence of an intervention effect on remission at 12 months.  **Further, as mentioned in the previous manuscript draft, the intervention effect sizes observed in the current study are comparable to the long-term outcomes achieved by more intensive interventions delivered intervention [1]. We have referenced another recent systematic review in the manuscript that reported similar effect sizes for 12-month follow-up for adolescent-focused interventions [2]. Given that the effect size of our brief, transdiagnostic intervention, delivered by trained lay-counsellor, is within range of other more intensive, specialized mental health interventions, our findings may warrant attention.**  References:   1. Weisz JR, Kuppens S, Ng MY, Eckshtain D, Ugueto AM, Vaughn-Coaxum R, et al. What five decades of research tells us about the effects of youth psychological therapy: A multilevel meta-analysis and implications for science and practice. Am Psychol. 2017 Mar;72(2):79–117. doi:10.1037/a0040360 2. Pilling S, Fonagy P, Allison E, Barnett P, Campbell C, Constantinou M, et al. Long-term outcomes of psychological interventions on children and young people’s mental health: A systematic review and meta-analysis. PLoS One. 2020 Nov 16;15(11):e0236525. |
| 18. | The authors said "we respectfully disagree that the study is not powered for the 12-month primary outcomes". However, it says in page 10 "The estimated sample size for the originally stipulated primary endpoint assumed loss to follow-up of 15% at six weeks (based on piloting) and a 1:1 allocation ratio", therefore clearly the sample size was calculated for outcome at 6 weeks rather than at 12 months. Now the primary analyses have changed to 12 months but the sample size was still from that at 6 weeks? Just to re-iterate, therefore, the study was not powered (sample size) for primary outcomes at 12 months. The sample size calculation at 6 weeks doesn't match the primary outcomes at 12 months. Due to the inadequte sample size, mostly borderline results and the 30% loss of follow-ups and 22% non-adherence to protocol (intervention arm), the reliability and believability of the results are subject to scrutiny | **We have acknowledged throughout the manuscript that this study is a post-hoc extension; thus, the sample size calculations were not powered for the observed long-term attrition rate. We recognize this as one of the study's main limitations, as outlined in the Abstract and Discussion sections. However, to reduce the risk of bias due to attrition, variables associated with missing were adjusted in the sensitivity analysis (Page 26, lines 429-432) and these findings were discussed on Page 35, lines 578-588.**  **Abstract (pages 4-5, lines 78-80, lines 86-88)**  The main limitations of the study's methodology were the lack of sample size calculations powered for 12-month follow-up and the absence of cost-effectiveness analyses using the primary outcomes.  … The findings need to be interpreted with caution, as this study was a post-hoc extension, and thus, the sample size calculations did not take into account the relatively high attrition rate observed during the long-term follow-up.  **Discussion (page 34, lines 561-571)**  First, the 12-month follow-up was a post-hoc addition to the originally scheduled follow-up assessments at six and 12 weeks. Consequently, the sample size calculation did not take into account the numbers expected for the 12-month assessments. The observed LTFU rate at the 12-month point (29.4%) was higher than assumed for the original endpoints. That said, the 12-month LTFU was similar to the attrition rate at long-term follow-up in other randomized trials of interventions for youth anxiety and depression [27,28]. To reduce risk of bias due to attrition while using complete-case analysis, variables associated with LTFU were adjusted in these analyses. Further, sensitivity analysis with missing data showed intervention effects were similar to the primary analysis for most variables except SDQ Total Difficulties and Impact scores at 12 months, where the effects were weaker. |
